# Supplementary material for: Proteomic profiling of MIS-C patients indicates heterogeneity relating to interferon gamma dysregulation and vascular endothelial dysfunction
Source: Nat Commun. 2021 Dec 10;12:7222. doi: 10.1038/s41467-021-27544-6 (PMC8664884; doi:10.1038/s41467-021-27544-6)
Supplement: Supplementary file 1 — Supplementary Information [file 41467_2021_27544_MOESM1_ESM.pdf]

Supplementary Materials

Supplementary Tables

Supplementary Table 1. Demographic and clinical information about patients with MIS-C, severe COVID-19 or minimal COVID-19.

Supplementary Table 2. Most extreme laboratory values during admission for patients with MIS-C, severe COVID-19 or minimal COVID-19.

Supplementary Table 3. Contingency Table of MAS by Disease Category.

Supplementary Table 4. Contingency Table of TMA by Disease Category.

Supplementary Table 5. Contingency tables for MIS-C patients in the IFNγ-high versus -low clusters and those who met criteria for TMA or MAS.

Supplementary Figures

Supplementary Figure 1. Validations of the Olink data set compared to previously published datasets.

Supplementary Figure 2. Principal Component Analysis (PCA) of proteins.

Supplementary Figure 3. Differentially expressed proteins between different disease states.

Supplementary Figure 4. Heterogeneity of disease phenotypes among MIS-C patients.

Supplementary Table S1. Demographic and clinical information about patients with MIS-C, severe COVID-19 or minimal COVID-19.

	MISC<br>N =22	Severe COVID-19<br>N =15	Minimal COVID-19<br>N = 26
**Age Median (IQR)**	9 (6.25-13)	16 (14.5-18)	14 (5.75-17)
**BMI Percentile Median (IQR)**	92.2 (80-97.5; N=21)	81.6 (66.1, 94.8; N=14)	73.4 (47.2, 93.8; N= 22)
**Gender N (%)**			
Female	10 (45)	7 (47)	12 (46)
Male	12 (55)	8 (53)	14 (54)
**Race N (%)**			
White	8 (36)	5 (33)	11 (42)
Black	11 (50)	5 (33)	12 (46)
Other	2 (9)	4 (27)	3 (12)
Declined	1 (5)	1 (7)	0 (0)
**Ethnicity N (%)**			
Hispanic (1)	3 (14)	4 (27)	4 (15)
Not Hispanic (2)	18 (82)	10 (67)	22 (85)
Declined (99)	1 (4)	1 (6)	0 (0)
**PICU Admission N (%)**			
Yes	17 (77)	15 (100)	21 (81)
No	5 (23)	0	5 (19)
**ECMO N (%)**			
Yes	0	2 (13)	0
No	22 (100)	13 (87)	26 (100)
**Inotropic Support N (%)**			
Yes	7 (32)	7 (47)	0
No	15 (68)	8 (53)	26 (100)
**Lymphopenic During Admission N (%)**			
Yes	18 (82)	9 (60)	14 (54)
No	4 (18)	6 (40)	12 (46)
**Neutropenic During Admission N (%)**			
Yes	0	5 (33)	8 (31)
No	22 (100)	10 (67)	18 (69)
**Previously Healthy N (%)**			
Yes	22 (100)	2 (13)	5 (19)
No	0	13 (87)	21 (81)
**Treatment at Time of First Sample**			
IVIG	0	-	-
Steroids	0	-	-
Both	14 (64)	-	-
None	8 (36)	-	-

Supplementary Table S2. Most extreme laboratory values during admission for patients with MIS-C, severe COVID-19 or minimal COVID-19.

**Value** (reference range)	**MIS-C**		**Severe COVID-19**		**Minimal COVID-19**	
	Median (IQR)	N	Median (IQR)	N	Median (IQR)	N
**COAGULATION**						
**D-Dimer, highest** (0.27 - 0.60 µg/ml FEU)	5.01 (4.01, 6.52)	21	1.06 (0.63, 5.4)	14	0.98 (0.583, 1.660	6
**PT, highest** (11.6-13.8 secs)	14.1 (14.5, 17.3)	22	14.7 (13, 17.9)	14	13.3 (12.3, 14.3)	13
**PTT, highest** (22-36 secs)	31.8 (29.5, 35.2)	21	47.2 (35.8, 57.9)	14	29.7 (27.1, 33.7)	13
**Fibrinogen, lowest** (172-471 mg/dL)	297 (230, 394)	21	303 (269, 478)	12	407 (262, 734)	6
**CHEMISTRY**						
**LDH, highest** (360-730 U/L)	767 (600,920)	21	966 (785,3160)	8	717 (575, 891)	6
**AST, highest** (15-45 U/L)	82.0 (66.0, 104)	22	115 (69.5, 293)	15	59.0 (40.0, 72.0)	21
**ALT, highest** (10-35 U/L)	62.0 (38.0, 87.8)	22	54.0 (32.5, 134)	15	30.0 (23.0, 47.0)	21
**Creatinine, highest** (0.3-0.8 mg/dL)	0.600 (0.525, 1.20)	22	0.5 (0.300, 1.20)	15	0.450 (0.300, 0.725)	24
**Bilirubin, highest** (0.6-1.4 mg/dL)	0.950 (0.600, 1.3)	22	0.700 (0.50, 0.95)	15	0.700 (0.400, 1.00)	21
**Sodium, lowest** (136-145 mmol/L)	131 (130, 135)	22	136 (133, 138)	15	136 (133, 137)	24
**HEMATOLOGY**						
**Neutrophils** (1,540 - 7,040 /uL)						
Lowest	7330 (5110, 8150)	22	2420 (1140, 3770)	11	3090 (1490, 3900)	26
Highest	13500 (11200, 21400)	22	7970 (6200, 13000)	11	5250 (3030, 8930)	26
**Lymphocytes, lowest,** (970-3,260 /uL)	610 (305, 878)	22	890 (390, 1380)	11	1220 (585, 2100)	26
**Hemoglobin, lowest** (12-16 g/dL)	8.45 (7.53, 9.53)	22	10.1 (7.30, 11.9)	11	9.75 (7.83, 12.1)	26
**Platelets, lowest** (150-400 K/µL)	150 (124, 189)	22	146 (86.5, 175)	15	208 (128, 291)	26
**INFLAMMATORY & CARDIAC**						
**Ferritin, highest** (10.0-82.0 ng/ml)	892 (665, 1370	21	217 (165, 1140)	13	1060 (307, 1470)	6
**CRP, highest** (0-0.9 mg/dL)	28.2 (19.2, 35.1	22	16.1 (5.75, 31.7	15	4.55 (1.30, 18.2)	16
**ESR, highest** (0-20 mm/hr)	68.0 (46.0, 103)	21	20.0 (13.0, 23.0)	5	46.5 (13.8, 85.5)	12
**BNP, highest** (≤100 pg/mL)	1060 (563, 1720)	21	223 (50.2, 454)	11	25.8 (10.0, 239)	4
**Troponin, highest** (<0.3 ng/ml)	0.600 (0.07, 1.87)	21	0.115 (0.02, 0.93)	12	0.01 (0.01, 0.015)	3

Supplementary Table S3 Contingency Table of MAS by Disease Category.

	MAS	No MAS
MIS-C	11	10
Minimal	3	3
Severe	9	4

Supplementary Table S4 Contingency Table of TMA by Disease Category.

	TMA	No TMA
MISC	7	8
Severe	5	3
Minimal	1	10

Supplementary Table S5. Contingency tables for MIS-C patients in the IFNγ-high versus -low clusters and those who met criteria for TMA or MAS.

TMA

	IFNγ-low	IFNγ-high
No TMA	5	3
TMA	6	1
%TMA	55%	25%

MAS

	IFNγ-low	IFNγ-high
No MAS	8	1
MAS	5	5
%MAS	38%	83%

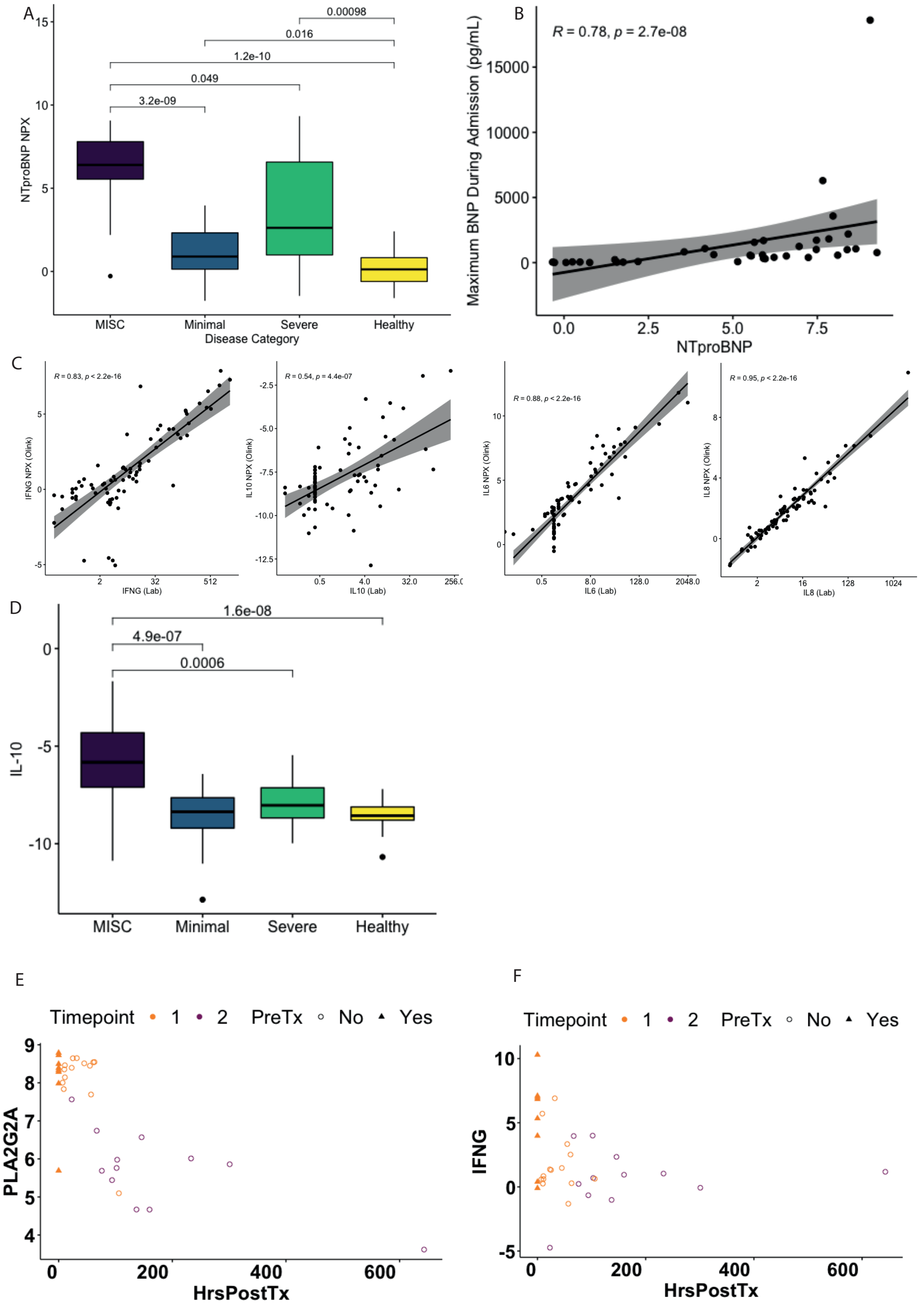

Supplementary Figure S1. Validations of the Olink data set compared to previously published datasets. (A) N-terminal prohormone brain natriuretic peptide (NTproBNP) levels compared between patients with Multisystem Inflammatory Syndrome in Children (MIS-C;

N=22), minimal SARS-CoV-2 infection (N=26), severe COVID-19 (N=15) and healthy controls (N=25). P-values computed with pairwise comparisons using Wilcoxon rank sum test following Kruskal-Wallis testing. Horizontal line represents median, with bounds of box representing interquartile range. Whiskers represent 1.5x the interquartile range. Dots represent outliers. (B) In patients on whom a brain-type natriuretic peptide (BNP) was measured during admission (N=36), these values were correlated with NTproBNP with a strong correlation. R values computed with Spearman correlation. Error bands represent 95% confidence interval. (C) Correlations between interferon gamma (IFNγ), interleukin-10 (IL-10), IL-6 and IL-8 measured by the Olink data set and by the clinical lab (N=76). R values computed with Spearman correlation. Error bands represent 95% confidence interval. (D) IL-10 levels between MIS-C (N=22) patients and patients with minimal SARS-CoV-2 infection (N=26; p=0.00000049), severe COVID-19 (N=15; p=0.0006) and healthy controls (N=25, p=0.000000016). P-values computed with pairwise comparisons using Wilcoxon rank sum test following Kruskal-Wallis testing. Horizontal line represents median, with bounds of box representing interquartile range. Whiskers represent 1.5x the interquartile range. Dots represent outliers. Decay in phospholipase A2 (PLA2G2A; E) and IFNγ; (F) over time for MIS-C patients is shown (N=22). Dots are colored by order of draw (first versus second timepoint). Shapes represent if samples were drawn prior to or after treatment with intravenous immune globulin (IVIG) or corticosteroids. All p-values were calculated using two-sided tests.

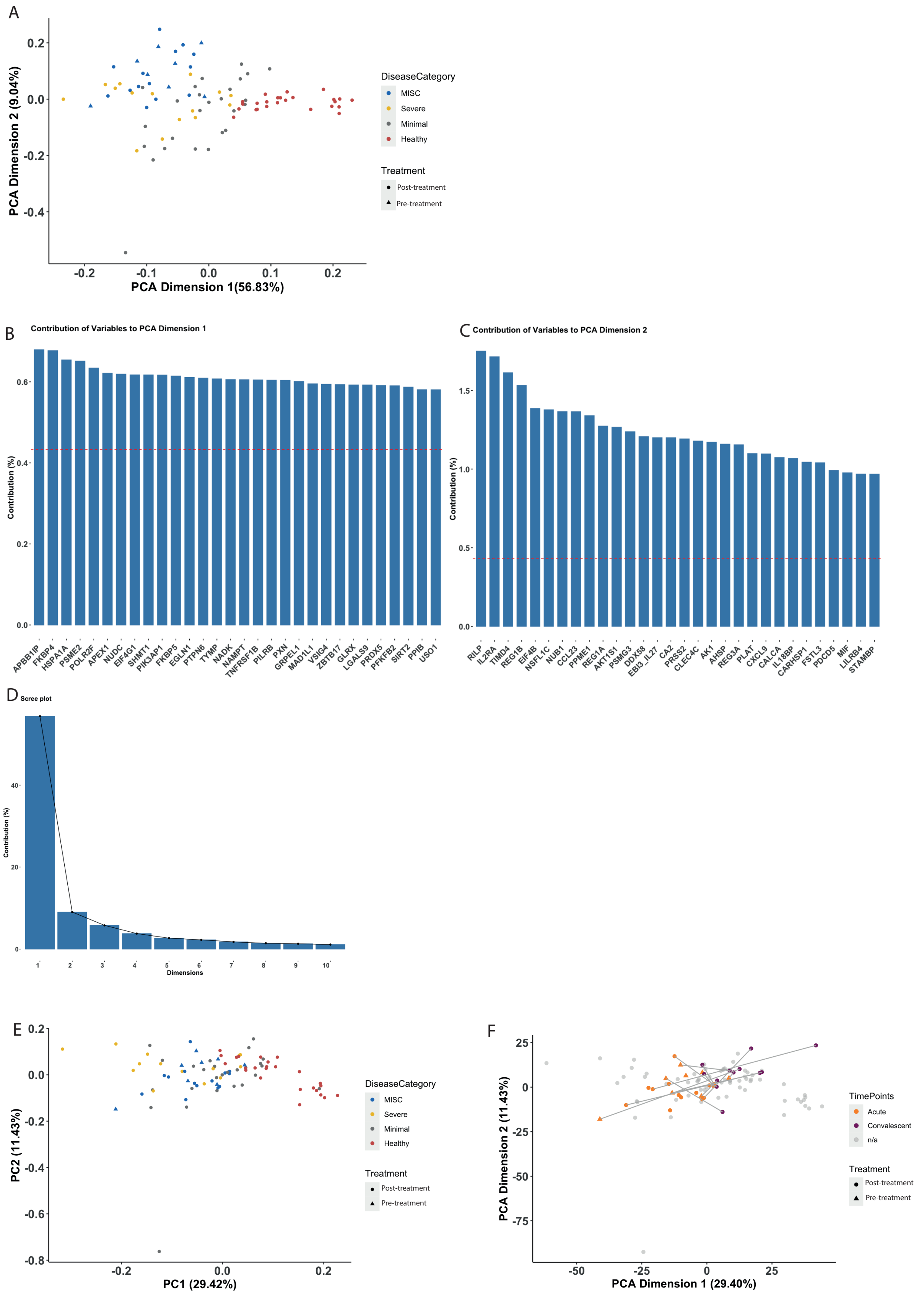

Supplementary Figure S2. Principal Component Analysis (PCA) of proteins. Clustering was examined using principal component analysis (PCA) of DEPs between all 4 patient groups,

log2 fold-change threshold of 2 and FDR threshold of 0.01 (A). Pre- and post-treatment samples for MIS-C patients are denoted by shape (triangle and circle respectively). Top 30 proteins that contribute to PCA Dimension 1 (B) and PCA Dimension 2 (C). Scree plot of eigenvalues of principal components of the PCA is shown in panel (D). To understand the change in the proteome over time in MIS-C patients we first created a PCA of all proteins for all disease categories (E). MIS-C patients with matched acute and convalescent samples (N=12) were then transformed onto the space (F). Acute samples are shown in orange and convalescent samples in purple. Lines connect matched pairs. Pre- and post-treatment samples for MIS-C patients are denoted by shape (triangle and circle respectively).

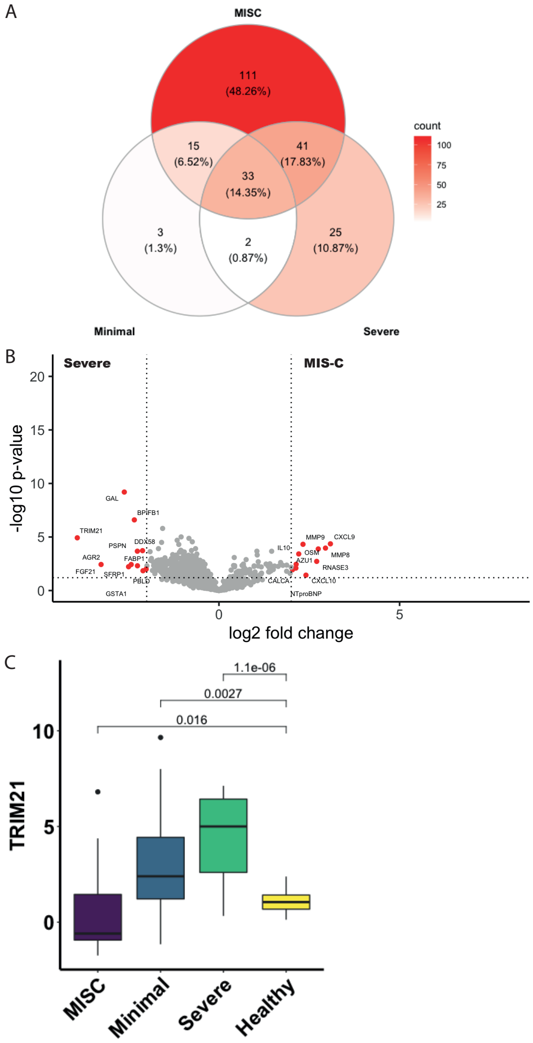

Supplementary Figure S3. Differentially expressed proteins between different disease states. (A) Venn diagram of overlap of differentially expressed proteins between different disease states and healthy patients. (B) Differentially expressed proteins between patients with Multisystem Inflammatory Syndrome in Children (MIS-C; N=22) and severe COVID-19 (N=15). Red dots represent proteins with a nominal p-value of less than 0.05 and a log2fold change of greater than 2. (C) TRIM21 levels among patients with MIS-C (N=22), Severe (N=15), Minimal disease (N=26) and healthy controls (N=25). P-values computed with pairwise comparisons using Wilcoxon rank sum test following Kruskal-Wallis testing.

Horizontal line represents median, with bounds of box representing interquartile range.

Whiskers represent 1.5x the interquartile range. Dots represent outliers.

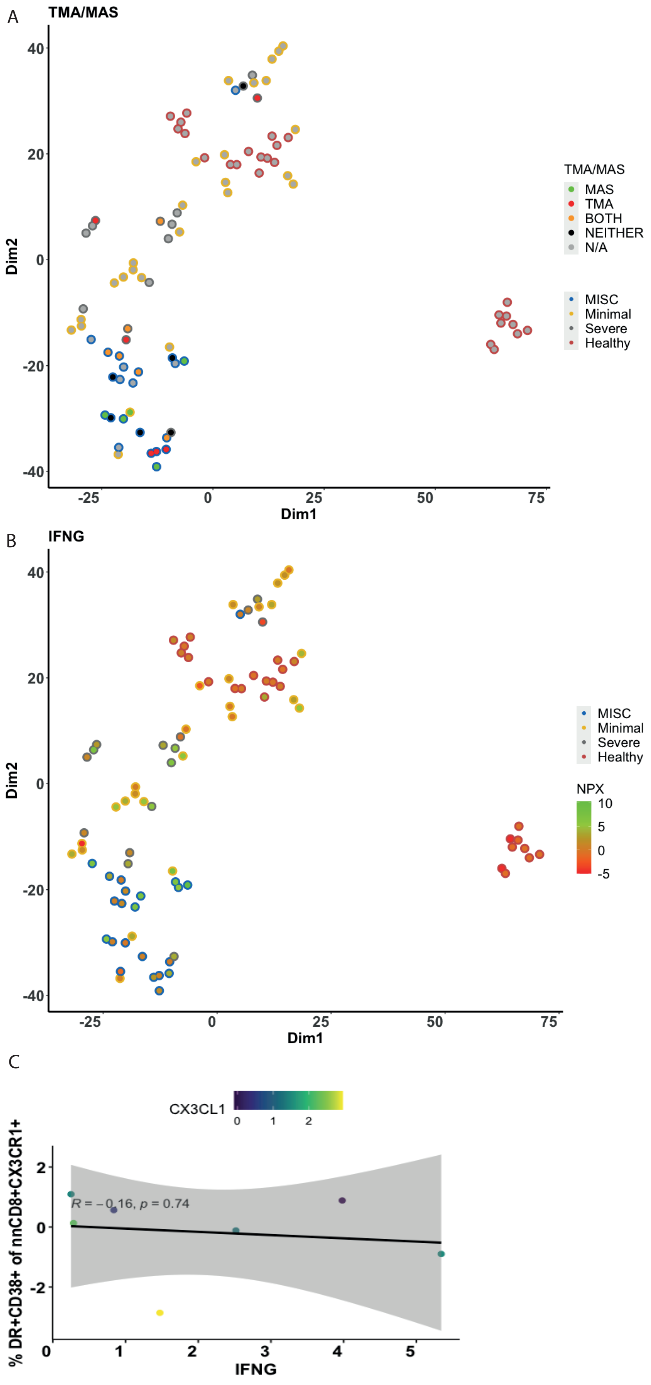

Supplementary Figure S4. Heterogeneity of disease phenotypes among MIS-C patients. In panels (A) t-distributed Stochastic Neighbor Embedding (tSNE) plot from Figure 1A are shown with dots colored by whether or not patients met criteria for thrombotic microangiopathy (TMA), macrophage activation syndrome (MAS), neither phenotype or both phenotypes. Rings around dots are colored by disease category, including Multisystem

Inflammatory Syndrome in Children (MIS-C; N=22), minimal SARS-CoV-2 infection (N=26), severe COVID-19 (N=15) and healthy controls (N=25). Panel (B) shows a similar output but with internal dots colored by interferon gamma (IFNγ) expression. Panel (C) demonstrates a lack of correlation between IFNγ level and percent DR+CD38+ non-naïve CD4+ CX3CR1+ T-cells in MIS-C patients (N=7). Dots colored by CX3CL1 expression. R value computed using Pearson's correlation coefficient after normality was demonstrated. All p-values were calculated using two-sided tests.

Supplementary Methods

```
---
title: "Olink_COVSars_Submission"
author: "Rawan Shraim"
date: "10/27/2021"
output: html_document
---

```{r setup, include=FALSE}
knitr::opts_chunk$set(echo = TRUE)
```

R Markdown

This is an R Markdown document. Markdown is a simple formatting syntax for
authoring HTML, PDF, and MS Word documents. For more details on using R
Markdown see .

When you click the **Knit** button a document will be generated that
includes both content as well as the output of any embedded R code chunks
within the document. You can embed an R code chunk like this:

```{r}
## Reading in Olink data

#Removing all initial variables
rm(list=ls())

#Reading in all needed libraries
library(dplyr)
library(ggfortify)
library(FactoMineR)
library(factoextra)
library(Rtsne)
library(ggrepel)
library(gridExtra)
library(ggpubr)
library(ggplot2)
library(reshape2)
library(viridis)
library(ggnewscale)


olink_data=read.table("~/Documents/Teachey lab/COV-
SARS/NPX_Wide_23FEB21.csv", sep=",", header=TRUE)

olink_data=read.table("NPXOnly_Olink.csv", sep=",", header=TRUE)

olink_clinical=read.delim("~/Box/Teachey_Lab/Projects/Proteomics COV-
SARS/Data/clindata_ino_NPX_2APR21.csv", sep = ",", header = T)

olink_clinical=read.delim("Meta_NPX_Olink.csv", sep = ",", header = T)

head(olink_data)
rownames(olink_data)=olink_data$SampleID
olink_data$SampleID=NULL

#Separating timepoint 1 from timepoint 2
tp1=olink_data[which(olink_data$Timepoint==1),]
head(tp1)
tp2=olink_data[which(olink_data$Timepoint==2),]
head(tp2)

```


```{r}
##PCA/tSNE for timepoint 1

#Selecting columns with NPX data only for PCA
tp1_pca=select(tp1, -(1:4))
head(tp1_pca)
pca_tp1_data=prcomp(tp1_pca, scale= TRUE)

## Abstracting presentation of variation in every PCA
fviz_eig(pca_tp1_data)

#Updating name for disease category
tp1$DiseaseCategory=tp1$DiseaseCat
tp1$DiseaseCategory[which(tp1$DiseaseCategory == "1")] = "MISC"
tp1$DiseaseCategory[which(tp1$DiseaseCategory == "2")] = "Minimal"
tp1$DiseaseCategory[which(tp1$DiseaseCategory == "3")] = "Severe"
tp1$DiseaseCategory[which(tp1$DiseaseCategory == "4")] = "Healthy"
head(tp1$DiseaseCategory)

#plotting pca of all disease categories at timepoint1
tp1$DiseaseCategory=factor(tp1$DiseaseCategory, levels = c("MISC",
"Severe", "Minimal", "Healthy"))

##Pre-treatment for MISC
preinf=read.delim("~/Box/Teachey_Lab/Projects/Proteomics COV-
```
```

```
SARS/Data/ALLMISC_TPS_5APR21.csv", sep = ",", header = T)
#creating shape vector
pretreatid=preinf$SubjectID[which(preinf$PreTx == 1)]
tp1$Treatment=rep("Post-treatment", nrow(tp1))
tp1$Treatment[which(tp1$SubjectID %in% pretreatid)]="Pre-treatment"

p=autoplot(pca_tp1_data, data=tp1, colour = "DiseaseCategory",
shape="Treatment")

p=p+ theme(plot.title = NULL, axis.title=element_text(size=14,
face="bold"),
        axis.text.x=element_text(size=14, face="bold"),
        axis.text.y=element_text(size=14,face="bold")) +
  theme(panel.grid.major = element_blank(), panel.grid.minor =
element_blank(),
        panel.background = element_blank(), axis.line.x.bottom =
          element_line(color="black"),axis.line.y.left =
element_line(color="black"))

set_palette(p, "jco")

#Tsne plots
library(Rtsne)
#Run tSNE
NPX_Data.tsne = Rtsne(tp1_pca, perplexity=3,max_iter=1000)

#Create tSNE dataframe for ggplot
TSNE.ggp.df =
data.frame(Dim1=NPX_Data.tsne$Y[,1],Dim2=NPX_Data.tsne$Y[,2],Disease_Cat =
tp1$DiseaseCategory, subject=tp1$SubjectID)

TSNE.ggp.df$Treatment=rep("Post-treatment", nrow(TSNE.ggp.df))

TSNE.ggp.df$Treatment[TSNE.ggp.df$subject %in%  pretreatid]="Pre-treatment"

#Plot tSNE with subject labels
TSNE.ggp.df$Disease_Cat <- factor(TSNE.ggp.df$Disease_Cat, levels =
c("MISC", "Severe", "Minimal", "Healthy"))

TSNE_old=read.delim("~/Box/Teachey_Lab/Projects/Proteomics COV-
SARS/Data/TSNE_map.csv", sep=",", header=T)

p=ggplot(TSNE.ggp.df,aes(x=Dim1, y=Dim2, col=Disease_Cat)) +
geom_point(aes(shape=Treatment), size = 3)+
  theme_bw() +  theme( axis.text.x=element_text(size=12, face="bold", color
= "black"),
                 axis.text.y=element_text (size=12, face="bold", color =
"black"),
                 panel.grid.major=element_blank(),
panel.grid.minor=element_blank(),
                 panel.background = element_rect(fill="white"),
                 axis.line = element_line(colour = "black", size = 1,
linetype =
                                            "solid"),
                 panel.border = element_blank(),
                 legend.text=element_text(size=10),
                 legend.title = element_text(size=10)) + labs(col = "") +
  theme(axis.title.x=element_blank(),
         axis.title.y = element_blank())


set_palette(p, "jco")

##Mean and Median for healthy patients TP1

healthy=tp1_pca[which(tp1$DiseaseCategory == "Healthy"),]

mean_healthy=colMeans(healthy, na.rm = T)
median_healthy=apply(healthy, 2, median)

healthy_stats=data.frame(mean=mean_healthy, median=median_healthy)
rownames(healthy_stats)=colnames(healthy)
```


```{r}
## PCA of timepoint 2

#Abstracting NPX values only
tp2_pca=select(tp2, -(1:4))
head(tp2_pca)
pca_tp2_data=prcomp(tp2_pca, scale=TRUE)

#Updating names for disease category
tp2$DiseaseCategory=tp2$DiseaseCat
tp2$DiseaseCategory[which(tp2$DiseaseCategory == "1")] = "MISC"
tp2$DiseaseCategory[which(tp2$DiseaseCategory == "2")] = "Minimal"
tp2$DiseaseCategory[which(tp2$DiseaseCategory == "3")] = "Severe"
tp2$DiseaseCategory[which(tp2$DiseaseCategory == "4")] = "Healthy"
head(tp2$DiseaseCategory)
```

```
autoplot(pca_tp2_data, data=tp2, colour = "DiseaseCategory")
```

```{r}
##PCA timepoint 1 and timepoint 2 for MISC patients only

#PCA for all disease groups TP1
g= autoplot(pca_tp1_data, data=tp1, colour = "DiseaseCategory")
g + theme(plot.title = NULL, axis.title=element_text(size=14, face="bold"),
          axis.text.x=element_text(size=14, face="bold"),
axis.text.y=element_text
          (size=14, face="bold")) +scale_color_manual(values=
c("#440154FF", "#31688EFF",

"#35B779FF", "#FDE725FF"), breaks = c("MISC", "Severe", "Minimal",
"Healthy")) +
  ylab("PCA Dimension 2 (11.43%)") +
  xlab("PCA Dimension 1 (29.40%)") +
  theme(panel.grid.major = element_blank(), panel.grid.minor =
element_blank(),
        panel.background = element_blank(), axis.line.x.bottom =
          element_line(color="black"),axis.line.y.left =
element_line(color="black"))


#Setting up data frames for MISC patients only for tp1 and tp2
misc_tp1_ind=which(tp1$DiseaseCategory == "MISC")
misc_tp2_ind=which(tp2$DiseaseCategory =="MISC")

#MISC timepoint 1 metadata and pca dataframes
misc_tp1_md=tp1[misc_tp1_ind,]
misc_tp1_pca=tp1_pca[misc_tp1_ind,]

#MISC timepoint 2 metadata and pca dataframes
misc_tp2_md=tp2[misc_tp2_ind,]
misc_tp2_pca=tp2_pca[misc_tp2_ind,]

#Transforimg timepoint 2 data points onto timepoint 1 space for MISC
pca_tp2_trans=scale(misc_tp2_pca, pca_tp1_data$center, pca_tp1_data$scale)
%*% pca_tp2_data$rotation

#Abstracting PCA1 and PCA2 from the tp1 PCA
pca_tp1_coord=pca_tp1_data$x

#Abstracting only the first 2 PCs into a separate dataframe
pca_tp1_coord=as.data.frame(pca_tp1_coord[,1:2])
pca_tp1_coord$timepoint=tp1$Timepoint
pca_tp1_coord$subjectid=tp1$SubjectID
pca_tp1_coord$diseasecat=tp1$DiseaseCategory

#Abstracting only the first 2 PCs for timepoint 2 into a separate dataframe
misc_tp2x=as.data.frame(pca_tp2_trans[,1:2])
misc_tp2x$timepoint=misc_tp2_md$Timepoint
misc_tp2x$subjectid=misc_tp2_md$SubjectID
misc_tp2x$diseasecat=misc_tp2_md$DiseaseCategory

#Combining dataframe of TP1 and TP2 into one
pca_comb2=rbind(pca_tp1_coord, misc_tp2x)

#Creating dataframe to input into ggplot
data_transformation=data.frame("pca1"=pca_comb2[,1], "pca2"=pca_comb2[,2],
"subject_id"=pca_comb2$subjectid, "timepoint"=pca_comb2$timepoint,
"diseasecat"=pca_comb2$diseasecat)

rownames(data_transformation)=rownames(pca_comb2)

#creating shape vector
pretreatid=preinf$SampleID[which(preinf$PreTx == 1)]
data_transformation$Treatment=rep("Post-treatment",
nrow(data_transformation))
data_transformation$Treatment[which(rownames(data_transformation) %in%
pretreatid)]="Pre-treatment"

#Creating the color vector for the time points
data.colors = rep("grey",nrow(data_transformation))
data.colors[misc_tp1_ind]="red"
data.colors[which(data_transformation$timepoint==2)]="blue"


#Updating names of timepoints for ggplot labeling
ind=which(data.colors=="grey")
data_transformation$timepoint[ind]="n/a"
tp1_ind=which(data_transformation$timepoint==1)
data_transformation$timepoint[tp1_ind]="Acute"
tp2_ind=which(data_transformation$timepoint==2)
data_transformation$timepoint[tp2_ind]="Convalescent"

data_transformation$timepoint=as.factor(data_transformation$timepoint)
#Creating color scheme for timepoints
cols=c("Acute"="#F98C0AFF","Convalescent"="#89226AFF", "n/a"="#BEBEBE80")
```

```
#Plotting datapoints from TP1 and TP2
ggplot(data_transformation, aes(x=pca1, y=pca2, col=timepoint,
group=subject_id))+
  geom_point(aes(shape=Treatment),size = 2) +
  scale_color_manual(values=cols, name="TimePoints") +
  geom_line(col="grey")+
  ylab("PCA Dimension 2 (11.43%)") +
  xlab("PCA Dimension 1 (29.40%)") +
  theme(plot.title = NULL, axis.title=element_text(size=14, face="bold"),
        axis.text.x=element_text(size=14, face="bold"),
        axis.text.y=element_text(size=14,face="bold")) +
  theme(panel.grid.major = element_blank(), panel.grid.minor =
element_blank(),
        panel.background = element_blank(), axis.line.x.bottom =
          element_line(color="black"),axis.line.y.left =
element_line(color="black"))

#Elements contributing to the PCA plot
fviz_pca_var(pca_tp1_data,
             col.var = "contrib",
             labelsize = 2,
             select = list(contrib=10),
             gradient.cols = NULL,
             repel = TRUE, max.overlaps=Inf) + theme_classic(base_size=3) +
labs(col.var="Contribution") + ylab("PCA Dimension 2 (11.43%)") + xlab("PCA
Dimension 1(29.40%)")

head(pca_tp1_data$scale)

#Calculate slopes for tp1 and tp2 changes in MISC patients

#Combine all data for misc in one dataframe
misc_alltp=data_transformation[which(data.colors=="blue" |
data.colors=="red"),]

#separate id's for patients who have complete data.
comp_pt=misc_alltp$subject_id[which(misc_alltp$timepoint=="2")]
#create an empty dataframe to hold the slope values
slopes_comp=data.frame(matrix(ncol=length(comp_pt)))
colnames(slopes_comp)=comp_pt

#loop through every patient to get the slope values and save in slopes_comp
dataframe
for (i in 1:length(comp_pt)){
  x=misc_alltp$pca1[which(misc_alltp$timepoint=="2" &
misc_alltp$subject_id==comp_pt[i])]-
misc_alltp$pca1[which(misc_alltp$timepoint=="1" &
misc_alltp$subject_id==comp_pt[i])]
  y = misc_alltp$pca2[which(misc_alltp$timepoint=="2" &
misc_alltp$subject_id==comp_pt[i])]-
misc_alltp$pca2[which(misc_alltp$timepoint=="1" &
misc_alltp$subject_id==comp_pt[i])]
  slope=y/x
  slopes_comp[1,i]=slope

}
```

```{r}
##Flow Cytometry Data for Lymphocytes

#Reading in the lymph flow data
lymph_flow=read.table("~/Box/Teachey_Lab/Projects/Proteomics COV-
SARS/Data/COVID_TP1_Olink_LymphFlow_3MAR21.csv", sep=",", header=TRUE)

#Reading in the lymph flow data
lymph_flow=read.table("LymphFlow_NPX.csv", sep=",", header=TRUE)

head(lymph_flow)
rownames(lymph_flow)=lymph_flow$SubjectID
head(lymph_flow)

#Selecting rows only for flow data
lymph2_flow=select(lymph_flow, matches("^perc"))
head(lymph2_flow)

#Removing rows with 0 variance to run the PCA
lymph2_flow_pca=lymph2_flow[,which(apply(lymph2_flow, 2, var) !=0)]
pca_lymph=prcomp(lymph2_flow_pca, scale=TRUE)

#Creating a column with categories
lymph_flow$DiseaseCategory=lymph_flow$DiseaseCat
lymph_flow$DiseaseCategory[which(lymph_flow$DiseaseCategory == "1")] =
"MISC"
lymph_flow$DiseaseCategory[which(lymph_flow$DiseaseCategory == "2")] =
"Minimal"
lymph_flow$DiseaseCategory[which(lymph_flow$DiseaseCategory == "3")] =
"Severe"
```

```
lymph_flow$DiseaseCategory[which(lymph_flow$DiseaseCategory == "4")] =
"Healthy"
head(lymph_flow$DiseaseCategory)


#Loading select markers from flow data
markers=read.table("~/Box/Teachey_Lab/Projects/Proteomics COV-
SARS/Data/markers.interest.csv", sep=",")

#Creating dataframe for only select markers from the flow data
markers_data=lymph_flow[,markers$V1]
markers_data$subject_id=lymph_flow$SubjectID
markers_data$disease_cat=lymph_flow$DiseaseCat
markers_data$disease_cat2=lymph_flow$DiseaseCategory
head(markers_data)

#Run tSNE
NPX_Data.tsne = Rtsne(tp1_pca, perplexity=3,max_iter=1000)

#Create tSNE dataframe for ggplot
TSNE.ggp.df =
data.frame(Dim1=NPX_Data.tsne$Y[,1],Dim2=NPX_Data.tsne$Y[,2],Disease_Cat =
tp1$DiseaseCategory)

#Function for plotting select marker data

marker_pca=function(marker_name){
  color.tsne=rep("",nrow(tp1))
  color.tsne[which(tp1$SubjectID %in%
lymph_flow$SubjectID)]=markers_data[,marker_name]
  color.tsne=as.numeric(color.tsne)
  alpha.tsne=rep(0.5,nrow(tp1))
  alpha.tsne=as.numeric(alpha.tsne)
  alpha.tsne[which(!is.na(color.tsne))] = 1

  p = ggplot(TSNE.ggp.df,aes(x=Dim1, y=Dim2)) + geom_point(aes(color =
color.tsne, alpha = alpha.tsne), size = 3) +
    scale_color_viridis(name ="%", option = "plasma") + xlab("") + ylab("")
+scale_alpha(range = c(0.5, 1)) + guides(alpha = FALSE)

  p=p + ggtitle(colnames(markers_data[marker_name]))
  p=p + theme(axis.title=element_text(size=12, face="bold"),
              axis.text.x=element_text(size=12, face="bold", color =
"black"),
              axis.text.y=element_text (size=12, face="bold", color =
"black"),
              panel.grid.major=element_blank(),
panel.grid.minor=element_blank(),
              panel.background = element_rect(fill="white"),
              axis.line = element_line(colour = "black", size = 1, linetype
= "solid"),
              panel.border = element_blank())

  c=ggplot(TSNE.ggp.df,aes(x=Dim1, y=Dim2, col=Disease_Cat)) +
geom_point()+
  theme_bw() + scale_color_viridis(discrete=TRUE)



  return(p)
}



#Loop through the marker numbers using the function
marker_pca(1)

##plotting select markers with different scales

marker_pca_diffscale=function(marker_name){
  color.tsne=rep("",nrow(tp1))
  color.tsne=tp1[,marker_name]
  color.tsne=as.numeric(color.tsne)

  p = ggplot(TSNE.ggp.df,aes(x=Dim1, y=Dim2)) +
    geom_point(aes(color=Disease_Cat, size = 5)) +
    scale_color_viridis(discrete=TRUE, name="") +
    new_scale("color") +
    geom_point(aes(color = color.tsne), size = 2.5) +
    scale_color_gradient(name ="NPX", low="Red", high="Green") +
    xlab("Dim1") + ylab("Dim2")  +
    guides(size = FALSE) +
    mytheme

  p=p + ggtitle(marker_name)
  return(p)
}
```


```{r}
```

```
##Correlation testing between NPX markers and Lymph flow markers

#normalizing lymph data function per column
normalize = function(dataset){
  norm_data=dataset
  for(i in 1:ncol(norm_data)){
    norm_data[,i]=norm_data[,i]/mean(norm_data[,i], na.rm = TRUE)
  }
  return(norm_data)
}
lymph_norm=normalize(lymph2_flow)
head(lymph_norm)
lymph_norm$disease_cat=lymph_flow$DiseaseCategory

#Creating correlation matrices

#Selecting on data columns
lymph_corr_data1=lymph_flow[,6:ncol(lymph_flow)]

#Selecting only NPX olink data
lymph_corr_data2=select(lymph_corr_data1, -matches("^perc"))
head(lymph_corr_data2)

#Selecting only flow data
lymph_corr_data3=select(lymph_corr_data1, matches("^perc"))

#Selecting only specific markers
markers_norm=lymph_norm[,which(names(lymph_norm) %in% markers$V1)]
markers_data=lymph_flow[,markers$V1]

#Creating an empty correlation matrix
corr_mat=data.frame(matrix(ncol = ncol(lymph_corr_data2), nrow =
ncol(markers_norm)), row.names = colnames(markers_norm))

corr_mat_allmarkers=data.frame(matrix(ncol = ncol(lymph_corr_data2), nrow =
ncol(lymph_corr_data3)), row.names = colnames(lymph_corr_data3))


# Correlation norm markers
for(i in 1:ncol(markers_norm)){
  for(r in 1:ncol(lymph_corr_data2)){
    corr_mat[i,r] = cor(markers_norm[,i], lymph_corr_data2[,r], method =
c("spearman"))
  }
}

# Correlation for all flow data
for(i in 1:ncol(lymph_corr_data3)){
  for(r in 1:ncol(lymph_corr_data2)){
    corr_mat_allmarkers[i,r] = cor(lymph_corr_data3[,i],
lymph_corr_data2[,r], method = c("spearman"))
  }
}

colnames(corr_mat)=colnames(lymph_corr_data2)
colnames(corr_mat_allmarkers)=colnames(lymph_corr_data2)

#Correlation cut off for normalized data
high_corr2=which(abs(corr_mat) >=0.6, arr.ind = TRUE)
olink_m2=names(corr_mat)[high_corr2[,2]]
flow_m2=rownames(corr_mat)[high_corr2[,1]]

#correlation cut off for all flow data
high_corr3=which(abs(corr_mat_allmarkers) >=0.8, arr.ind = TRUE)
olink_m3=names(corr_mat_allmarkers)[high_corr3[,2]]
flow_m3=rownames(corr_mat_allmarkers)[high_corr3[,1]]

high_corr2_df=data.frame("flow_marker"=flow_m2, "olink_markers"=olink_m2,
"correlation"=corr_mat[high_corr2])
high_corr3_df=data.frame("flow_marker"=flow_m3, "olink_markers"=olink_m3,
"correlation"=corr_mat_allmarkers[high_corr3])

# Significance testing correlation
correlation_table=data.frame(matrix(nrow=nrow(high_corr3), ncol = 4))
colnames(correlation_table)= c("marker", "olink","corr_val","p_val")

for (i in 1:nrow(high_corr3)){
  a=cor.test(lymph_corr_data3[,high_corr3[i,1]],
lymph_corr_data2[,high_corr3[i,2]], method = "spearman", exact = F)
  correlation_table[i,1]=names(lymph_corr_data3)[high_corr3[i,1]]
  correlation_table[i,2]=names(lymph_corr_data2)[high_corr3[i,2]]
  correlation_table[i,3]=a$estimate
  correlation_table[i,4]=a$p.value

}
```


```{r}
##Tsne plots for lymph flow data
```

```
#Run tSNE
NPX_Data.tsne = Rtsne(lymph2_flow, perplexity=3,max_iter=1000)

#Create tSNE dataframe for ggplot
TSNE.ggp.df =
data.frame(Dim1=NPX_Data.tsne$Y[,1],Dim2=NPX_Data.tsne$Y[,2],Disease_Cat =
lymph_flow$DiseaseCategory)
#Plot tSNE
ggplot(TSNE.ggp.df,aes(x=Dim1, y=Dim2, col=Disease_Cat)) + geom_point()+
  theme_bw()
```

```{r}
## Differential Expression analysis

#DE between disease categories. P-val using FDR and threshold 0.01
de_groups = function(group1,group2){
  diff_exp_matrix_fc=apply(tp1_pca,MARGIN=2,function(X)
    mean(X[which(tp1$DiseaseCat==group1)])-
mean(X[which(tp1$DiseaseCat==group2)]))
  diff_exp_matrix_pval=apply(tp1_pca,MARGIN=2,function(X)

p.adjust(t.test(X[which(tp1$DiseaseCat==group1)],X[which(tp1$DiseaseCat==group2)])$p.value,method="BH",n=length(5:(ncol(tp1)
-1))))
  #filtering based on pval
  Pval_Threshold1 = 0.01
  FC_Threshold = 2
  Sig_text = names(diff_exp_matrix_pval)[which(diff_exp_matrix_pval<=
Pval_Threshold1)]
  #Filtering based on FC
  FC_text=names(diff_exp_matrix_fc)[which(abs(diff_exp_matrix_fc)>=
FC_Threshold)]
  DE_Genes=intersect(Sig_text, FC_text)
  de_fc=diff_exp_matrix_fc[DE_Genes]
  de_pval=diff_exp_matrix_pval[DE_Genes]
  final_mat=data.frame(
    "prot.name"= DE_Genes,
    "fc"= de_fc,
    "Pval"= de_pval
  )
  return(final_mat)

}

#DE between disease categories. Nominal p-value and threshold of 0.05
de_nompval = function(group1,group2){
  diff_exp_matrix_fc=apply(tp1_pca,MARGIN=2,function(X)
    mean(X[which(tp1$DiseaseCat==group1)])-
mean(X[which(tp1$DiseaseCat==group2)]))
  diff_exp_matrix_pval=apply(tp1_pca,MARGIN=2,function(X)

t.test(X[which(tp1$DiseaseCat==group1)],X[which(tp1$DiseaseCat==group2)])$p.value
)
  #filtering based on pval
  Pval_Threshold1 = 0.05
  FC_Threshold = 2
  Sig_text = names(diff_exp_matrix_pval)[which(diff_exp_matrix_pval<=
Pval_Threshold1)]
  #Filtering based on FC
  FC_text=names(diff_exp_matrix_fc)[which(abs(diff_exp_matrix_fc)>=
FC_Threshold)]
  DE_Genes=intersect(Sig_text, FC_text)
  de_fc=diff_exp_matrix_fc[DE_Genes]
  de_pval=diff_exp_matrix_pval[DE_Genes]
  final_mat=data.frame(
    "prot.name"= DE_Genes,
    "fc"= de_fc,
    "Pval"= de_pval
  )
  return(final_mat)

}

#Create tables of DE between disease categories. Using FDR cutoff
DE_1.2=de_groups("1","2")
DE_1.3=de_groups("1","3")
DE_1.4=de_groups("1","4")
DE_2.3=de_groups("2","3")
DE_2.4=de_groups("2","4")
DE_3.4=de_groups("3","4")



#Create tables for DE between disease categories. Using nominal p-val
cutoff

DE_1.3_NP=de_nompval("1","3")
DE_2.3_NP=de_nompval("2","3")

round_order=function(de_table){
```

```
  de_table$fc=round(de_table$fc, 0)
  table_ordered=de_table[order(de_table$fc, decreasing = T),]
  return(table_ordered)
}


DE_1.2=round_order(DE_1.2)
DE_1.3=round_order(DE_1.3)
DE_1.4=round_order(DE_1.4)
DE_2.3=round_order(DE_2.3)
DE_2.4=round_order(DE_2.4)
DE_3.4=round_order(DE_3.4)
DE_1.3_NP=round_order(DE_1.3_NP)
DE_2.3_NP=round_order(DE_2.3_NP)


#Compiling differentially expressed genes between all disease categories
de_all.rep=c(DE_1.2$prot.name,DE_1.3$prot.name, DE_1.4$prot.name,
DE_2.3$prot.name, DE_2.4$prot.name, DE_3.4$prot.name)
de_all.unique=unique(de_all.rep)

#venn diagram looking at overlapping genes

DEPVenn = list (MISC = DE_1.4$prot.name, Minimal = DE_2.4$prot.name, Severe
= DE_3.4$prot.name)
ggVennDiagram(DEPVenn, label_alpha = 0)
```

```{r}
## PCA and tSNE of DE genes between all Disease categories
tp1_de=tp1[,de_all.unique]
head(tp1_de)

tp1_de_pca=prcomp(tp1_de, scale=TRUE)

g=autoplot(tp1_de_pca, data=tp1, colour="DiseaseCategory",
shape="Treatment")
g=g +  theme(plot.title = NULL, axis.title=element_text(size=14,
face="bold"),
         axis.text.x=element_text(size=14, face="bold"),
axis.text.y=element_text
         (size=14,face="bold")) + scale_color_manual(values=
c("#440154FF", "#31688EFF",

"#35B779FF", "#FDE725FF"),
                                                     breaks = c("MISC",
"Severe",
                                                                "Minimal",
"Healthy")) +
  ylab("PCA Dimension 2 (9.04%)") +
  xlab("PCA Dimension 1(56.83%)") +
  theme(panel.grid.major = element_blank(), panel.grid.minor =
element_blank(),
        panel.background = element_blank(), axis.line.x.bottom =
          element_line(color="black"),axis.line.y.left =
element_line(color="black"))
set_palette(g, "jco")
## Eigen values
fviz_eig(tp1_de_pca)
fviz_pca_var(tp1_de_pca)


eigenval=get_eig(tp1_de_pca)
head(eigenval)
var=get_pca_var(tp1_de_pca)
var_contrib=var$contrib
#get top 20 contributing factors to the pca clustering
top20_pc1=var_contrib[order(var_contrib[,1], decreasing = TRUE)[1:20],1]
top20_pc2=var_contrib[order(var_contrib[,2], decreasing = TRUE)[1:20],1]

fviz_pca_var(tp1_de_pca,
             labelsize = 4,
             select = list(contrib=10),
             gradient.cols = NULL,
             repel = TRUE, max.overlaps=Inf) + theme3 + ylab("PCA Dimension
2 (9.04%)") + xlab("PCA Dimension 1(56.83%)")+
             nogrid +
             theme(axis.line.x.bottom =
element_line(color="black"),axis.line.y.left = element_line(color="black"))


#Run tSNE
NPX_Data.tsne = Rtsne(tp1_de, perplexity=3,max_iter=1000)

#Create tSNE dataframe for ggplot
TSNE.ggp.df =
data.frame(Dim1=NPX_Data.tsne$Y[,1],Dim2=NPX_Data.tsne$Y[,2],Disease_Cat =
tp1$Diseasecat2)
#Plot tSNE
ggplot(TSNE.ggp.df,aes(x=Dim1, y=Dim2, col=Disease_Cat)) + geom_point()+
```

~~~~
  theme_bw()
```


```{r}
## Volcano plot generation

#Volcano plot for nominal p-value with 0.05 threshold
Create_VolPlot_np = function(group1,group2){
 de_genes=de_groups(group1,group2)
 #calculate fold change
 diff_exp_matrix_fc=apply(tp1_pca,MARGIN=2,function(X)
   mean(X[which(tp1$DiseaseCat==group1)])-
mean(X[which(tp1$DiseaseCat==group2)]))
 #calculate pval
 diff_exp_matrix_pval=apply(tp1_pca,MARGIN=2,function(X)

t.test(X[which(tp1$DiseaseCat==group1)],X[which(tp1$DiseaseCat==group2)])$p.value
)

 #Create dataframe from  plot in ggplot
 DIFF_Exp_data_df= data.frame(
   "log2FC" = diff_exp_matrix_fc,
   "-log10pval"= -log10(diff_exp_matrix_pval))

 #Plot  plot
 #Note to change text font size, change the 'size' vale in geom_text
function below
 Pval_Threshold = 0.05
 FC_Threshold = 2

 #creating the array for the colors in the  plot
 PVAL_Scores.colors = rep("grey",length(diff_exp_matrix_pval))
 PVAL_Scores.colors[which(diff_exp_matrix_pval<= Pval_Threshold &
abs(diff_exp_matrix_fc)>= FC_Threshold)] = "red"

 #creating labels to include on  plot
 PVAL_text = rep("",length(diff_exp_matrix_pval))
 PVAL_text[which(diff_exp_matrix_pval<= Pval_Threshold &
abs(diff_exp_matrix_fc)>= FC_Threshold)] =
names(which(diff_exp_matrix_pval<= Pval_Threshold &
abs(diff_exp_matrix_fc)>= FC_Threshold))

 Final_PLOT = ggplot(DIFF_Exp_data_df,aes(x=log2FC, y=X.log10pval)) +
   geom_point(color=PVAL_Scores.colors)+
   theme_classic(base_size = 15) +
   xlim(-4,8) +
   ylim(0,21) +
   geom_vline(xintercept=2, linetype="dotted", color="black", size=0.5) +
   geom_vline(xintercept=-2, linetype="dotted", color="black", size=0.5) +
   geom_hline(yintercept=1.2, linetype="dotted", color="black", size=0.5) +
   geom_text_repel(
      label=PVAL_text, size=3, color="black", min.segment.length = Inf,
max.overlaps = Inf
    ) + ylab("-log10 p-value") + xlab("log2 fold change")

 return(Final_PLOT)

}

Create_VolPlot_np("1","3") + ggtitle("Severe vs. MIS-C COVID-19")
Create_VolPlot_np("2","3") + ggtitle("Severe vs. Minimal COVID-19")

# Volcano plot for adjusted p-value with 0.01 threshold

Create_VolPlot_pad = function(group1,group2){
 de_genes=de_groups(group1,group2)
 #calculate fold change
 diff_exp_matrix_fc=apply(tp1_pca,MARGIN=2,function(X)
   mean(X[which(tp1$DiseaseCat==group1)])-
mean(X[which(tp1$DiseaseCat==group2)]))
 #calculate pval
 diff_exp_matrix_pval=apply(tp1_pca,MARGIN=2,function(X)

p.adjust(t.test(X[which(tp1$DiseaseCat==group1)],X[which(tp1$DiseaseCat==group2)])$p.value,metho
d
            ="BH",n=length(5:(ncol(tp1)-1))))

 #Create dataframe from  plot in ggplot
 DIFF_Exp_data_df= data.frame(
   "log2FC" = diff_exp_matrix_fc,
   "-log10pval"= -log10(diff_exp_matrix_pval))

 #Plot  plot
 #Note to change text font size, change the 'size' vale in geom_text
function below
 Pval_Threshold = 0.01
 FC_Threshold = 2

 #creating the array for the colors in the  plot
 PVAL_Scores.colors = rep("grey",length(diff_exp_matrix_pval))
~~~~

```
 PVAL_Scores.colors[which(diff_exp_matrix_pval<= Pval_Threshold &
abs(diff_exp_matrix_fc)>= FC_Threshold)] = "red"

 #creating labels to include on  plot
 PVAL_text = rep("",length(diff_exp_matrix_pval))
 PVAL_text[which(diff_exp_matrix_pval<= Pval_Threshold &
abs(diff_exp_matrix_fc)>= FC_Threshold)] =
names(which(diff_exp_matrix_pval<= Pval_Threshold &
abs(diff_exp_matrix_fc)>= FC_Threshold))

 Final_PLOT = ggplot(DIFF_Exp_data_df,aes(x=log2FC, y=X.log10pval)) +
   geom_point(color=PVAL_Scores.colors)+
   theme_classic(base_size = 15) +
   xlim(-4,8) +
   ylim(0,21) +
   geom_vline(xintercept=2, linetype="dotted", color="black", size=0.5) +
   geom_vline(xintercept=-2, linetype="dotted", color="black", size=0.5) +
   geom_hline(yintercept=2, linetype="dotted", color="black", size=0.5) +
   geom_text_repel(
      label=PVAL_text, size=3, color="black", min.segment.length = Inf,
max.overlaps = Inf
    ) + ylab("-log10 p-value") + xlab("log2 fold change")

 return(Final_PLOT)

}

Create_VolPlot_pad("1","4") + ggtitle("Healthy vs. MIS-C")
Create_VolPlot_pad("2","4") + ggtitle("Healthy vs. Minimal")
Create_VolPlot_pad("3","4") + ggtitle("Healthy vs. Severe")
```

```{r}
##Volcano plot for tp1 and tp2 for the following MISC patients - CD26,
CD48, CD50, CD55, and CD72

subjects=c("CD26", "CD48", "CD50", "CD55",  "CD72")

#Selecting indices for the subjects above in tp1 and tp2 data
ind_tp1=which(misc_tp1_md$SubjectID %in% subjects)
ind_tp2=which(misc_tp2_md$SubjectID %in% subjects)

#Create metadata dataframe for the select subjects
misc_select_tp1_md=misc_tp1_md[ind_tp1,]
misc_select_tp2_md=misc_tp2_md[ind_tp2,]

#Create PCA dataframe for the select subjects
misc_select_tp1_pca=misc_tp1_pca[ind_tp1,]
misc_select_tp2_pca=misc_tp2_pca[ind_tp2,]

misc_select_md=rbind(misc_select_tp1_md, misc_select_tp2_md)
misc_select_pca=rbind(misc_select_tp1_pca, misc_select_tp2_pca)


##Volcano plot for timepoint 1 vs timepoint 2
Create_VolPlot_misc = function(group1,group2){
 #calculate fold change
 diff_exp_matrix_fc=apply(misc_select_pca,MARGIN=2,function(X)
   mean(X[which(misc_select_md$Timepoint==group1)])-
mean(X[which(misc_select_md$Timepoint==group2)]))
 #calculate pval
 diff_exp_matrix_pval=apply(misc_select_pca,MARGIN=2,function(X)

t.test(X[which(misc_select_md$Timepoint==group1)],X[which(misc_select_md$Timepoint==group2)])$p.value
)

 #Create dataframe from  plot in ggplot
 DIFF_Exp_data_df= data.frame(
   "log2FC" = diff_exp_matrix_fc,
   "-log10pval"= -log10(diff_exp_matrix_pval))

 #Plot  plot
 #Note to change text font size, change the 'size' vale in geom_text
function below
 Pval_Threshold = 0.05
 FC_Threshold = 2

 #creating the array for the colors in the  plot
 PVAL_Scores.colors = rep("grey",length(diff_exp_matrix_pval))
 PVAL_Scores.colors[which(diff_exp_matrix_pval<= Pval_Threshold &
abs(diff_exp_matrix_fc)>= FC_Threshold)] = "red"

 #creating labels to include on  plot
 PVAL_text = rep("",length(diff_exp_matrix_pval))
 PVAL_text[which(diff_exp_matrix_pval<= Pval_Threshold &
abs(diff_exp_matrix_fc)>= FC_Threshold)] =
names(which(diff_exp_matrix_pval<= Pval_Threshold &
abs(diff_exp_matrix_fc)>= FC_Threshold))

 Final_PLOT = ggplot(DIFF_Exp_data_df,aes(x=log2FC, y=X.log10pval)) +
```

```
   geom_point(color=PVAL_Scores.colors)+
   theme_classic(base_size = 15) +
   xlim(-4,8) +
   ylim(0,10) +
   geom_vline(xintercept=2, linetype="dotted", color="black", size=0.5) +
   geom_vline(xintercept=-2, linetype="dotted", color="black", size=0.5) +
   geom_hline(yintercept=1.3, linetype="dotted", color="black", size=0.5) +
   geom_text_repel(
      label=PVAL_text, size=3, color="black", min.segment.length = Inf,
max.overlaps = Inf
    ) + ylab("-log10 p-value") + xlab("log2 fold change")

 return(Final_PLOT)

}

Create_VolPlot_misc("1","2") + ggtitle("MIS-C TP1 vs. MIS-C TP2")


#Create function for differential expression analysis for between
timepoints. Threshold 0.05 of nominal p-val

de_tp = function(group1,group2){
  diff_exp_matrix_fc=apply(misc_select_pca,MARGIN=2,function(X)
    mean(X[which(misc_select_md$Timepoint==group1)])-
mean(X[which(misc_select_md$Timepoint==group2)]))
  diff_exp_matrix_pval=apply(misc_select_pca,MARGIN=2,function(X)

t.test(X[which(misc_select_md$Timepoint==group1)],X[which(misc_select_md$Timepoint==group2)]
)
    $p.value)
  Pval_Threshold1 = 0.05
  FC_Threshold = 2
  Sig_text = names(diff_exp_matrix_pval)[which(diff_exp_matrix_pval<=
Pval_Threshold1)]
  #Filtering based on FC
  FC_text=names(diff_exp_matrix_fc)[which(abs(diff_exp_matrix_fc)>=
FC_Threshold)]
  DE_Genes=intersect(Sig_text, FC_text)
  de_fc=diff_exp_matrix_fc[DE_Genes]
  de_pval=diff_exp_matrix_pval[DE_Genes]
  final_mat=data.frame(
    "prot.name"= DE_Genes,
    "fc"= de_fc,
    "Pval"= de_pval
  )
  return(final_mat)
}

de_misc_tp=de_tp("1","2")
```

```{r}
##SC5B9 Genes expression lineplot

MISC_TPS=olink_data[which(olink_data$DiseaseCat ==1),]
misc_pts=unique(MISC_TPS$SubjectID)
keep_pts=data.frame()
for(i in 1:length(misc_pts)){
  n_tp=length(which(misc_pts[i] == MISC_TPS$SubjectID))
  if (n_tp >1){
    keep_pts=rbind(keep_pts, misc_pts[i])
  }else{next}
}

MISC_TPS=MISC_TPS[which(MISC_TPS$SubjectID %in% keep_pts$X.CD18.),]

MISC_TPS$X=NULL
MISC_TPS$SampleID=NULL

#Set TMA Gene Vector
TMA_Comprehensive_Panel = c("PLA2G2A", "PDGFC", "SELE", "CALCA", "NOS3",
"VWA1", "TYMP")

#Create Matrices with just TMA Genes and Subject ID, Disease Category, and
Timepoint. (It's not necessary to keep the disease category and time point
but I did anyways)
NPX_Orig.TMA.healthy =
olink_data[which(olink_data$DiseaseCat==4),c(3,4,5,which(colnames(olink_data
) %in% TMA_Comprehensive_Panel))]
MISC_TPS.TMA = MISC_TPS[,c(1,2,3,which(colnames(MISC_TPS) %in%
TMA_Comprehensive_Panel))]


#Use Melt function in Reshape function to transform matrices into GGPLOT
compatible matrices
MISC_TPS.TMA.melted = melt(MISC_TPS.TMA, id =
c("SubjectID","Timepoint","DiseaseCat") )
NPX_Orig.TMA.healthy.melted = melt(NPX_Orig.TMA.healthy, id =
c("SubjectID","Timepoint","DiseaseCat") )
```

```
colnames(MISC_TPS.TMA.melted) =
c("Patient","Timepoint","diseaseCat","Gene","NPX")
colnames(NPX_Orig.TMA.healthy.melted) =
c("Patient","Timepoint","diseaseCat","Gene","NPX")



#Create custom X axis points for genes (will be needed for the individual
line plots per patient per gene)... This puts the X coordinates in the
right order of how you would want the data show according to the order of
the genes in the initial 'TMA_Comprehensive_Panel' vector declared in the
beginning of the script
X.1 = as.numeric(sapply(unique(MISC_TPS.TMA.melted$Gene),function(X) rep(c(
which(TMA_Comprehensive_Panel== X) -0.2, which(TMA_Comprehensive_Panel ==
X) +0.2),length(unique(MISC_TPS.TMA.melted$Patient)))))
X.1.boxplot =
as.numeric(sapply(unique(NPX_Orig.TMA.healthy.melted$Gene),function(X) rep(
which(TMA_Comprehensive_Panel == X)
,length(unique(NPX_Orig.TMA.healthy.melted$Patient)))))




#Attach X asis points to ggplot matrices
MISC_TPS.TMA.melted.X.1 = cbind(MISC_TPS.TMA.melted,X.1)
NPX_Orig.TMA.healthy.melted.X.1 =
cbind(NPX_Orig.TMA.healthy.melted,X.1.boxplot)
MISC_TPS.TMA.melted.X.1$Timepoint[which(MISC_TPS.TMA.melted.X.1$Timepoint=="1")]="Acute
"
MISC_TPS.TMA.melted.X.1$Timepoint[which(MISC_TPS.TMA.melted.X.1$Timepoint=="2")]="Covalescent
"

#Change Colnames ... just because I wanted to
colnames(MISC_TPS.TMA.melted.X.1) =
c("Patient","Timepoint","diseaseCat","Gene","NPX","Xcoord")
colnames(NPX_Orig.TMA.healthy.melted.X.1) =
c("Patient","Timepoint","diseaseCat","Gene","NPX","Xcoord")

# Make sure the factor levels of the the genes are in desired ordered
MISC_TPS.TMA.melted.X.1$Gene <- factor(MISC_TPS.TMA.melted.X.1$Gene,levels
= TMA_Comprehensive_Panel)
NPX_Orig.TMA.healthy.melted.X.1$Gene <-
factor(NPX_Orig.TMA.healthy.melted.X.1$Gene,levels =
TMA_Comprehensive_Panel)

#Create blank ggplot for data
PLOT.PLINE = ggplot(MISC_TPS.TMA.melted.X.1,aes(y = NPX, x=Xcoord))

#Add boxplot first so it aears behind the line plots
PLOT.PLINE = PLOT.PLINE + geom_boxplot(data =
NPX_Orig.TMA.healthy.melted.X.1,aes(y = NPX, x=Xcoord, fill=Gene),
outlier.shape=NA) + scale_fill_manual(values=rep(c("#BEBEBE80"),7)) +
theme(legend.position = "none")

##generate stats for every group
library(ggpubr)
anno_df = compare_means(NPX ~ Timepoint, group.by = "Gene", data =
MISC_TPS.TMA.melted.X.1, method="wilcox.test",paired=TRUE)

#Loop through each gene to add patient data per gene (this will prevent the
formation of a continuous line across the entire plot for each patient)

for(GENE in TMA_Comprehensive_Panel)
{
  PLOT.PLINE = PLOT.PLINE + geom_line(data =
MISC_TPS.TMA.melted.X.1[which(MISC_TPS.TMA.melted.X.1$Gene==GENE),],
mapping = aes(y = NPX, x=Xcoord, color=Patient))

  PLOT.PLINE = PLOT.PLINE +
scale_color_manual(values=rep(c("#000000"),length(X.1)))


  PLOT.PLINE = PLOT.PLINE + geom_point(data =
MISC_TPS.TMA.melted.X.1[which(MISC_TPS.TMA.melted.X.1$Gene==GENE),],
mapping = aes(y = NPX, x=Xcoord, color=Patient, shape=Timepoint))

  pval=anno_df$p.format[which(anno_df$Gene==GENE)]
  xval=MISC_TPS.TMA.melted.X.1[which(MISC_TPS.TMA.melted.X.1$Gene==GENE &
                                      MISC_TPS.TMA.melted.X.1$Timepoint ==
                                      "Acute"),"Xcoord"]

  PLOT.PLINE = PLOT.PLINE + geom_text(x=xval[1], y=12, label=pval, size=3)

}



PLOT.PLINE
#Change other features of plot
PLOT.PLINE = PLOT.PLINE + guides(color=FALSE, fill=FALSE )
```

```
PLOT.PLINE = PLOT.PLINE + theme_classic()
PLOT.PLINE = PLOT.PLINE + ylim(-5,13)
#Conveert X-axis to Gene Names: Be sure the factor order for the ggplot
matches the the order of genes in 'labels' vecotr
PLOT.PLINE = PLOT.PLINE + scale_x_continuous(breaks = 1:7, labels =
TMA_Comprehensive_Panel)

PLOT.PLINE = PLOT.PLINE + theme(axis.text.x = element_text(angle = 90,
vjust = 0.5, hjust = 1))

PLOT.PLINE = PLOT.PLINE + theme(axis.title.x = element_blank())
plot(PLOT.PLINE)

```

```{r}
##SC5B9 Genes expression lineplot

MISC_TPS=olink_data[which(olink_data$DiseaseCat ==1),]
misc_pts=unique(MISC_TPS$SubjectID)
keep_pts=data.frame()
for(i in 1:length(misc_pts)){
  n_tp=length(which(misc_pts[i] == MISC_TPS$SubjectID))
  if (n_tp >1){
    keep_pts=rbind(keep_pts, misc_pts[i])
  }else{next}
}

MISC_TPS=MISC_TPS[which(MISC_TPS$SubjectID %in% keep_pts$X.CD18.),]

MISC_TPS$X=NULL
MISC_TPS$SampleID=NULL

#Set TMA Gene Vector
TMA_Comprehensive_Panel = c("PLA2G2A", "PDGFC", "SELE", "CALCA", "NOS3",
"VWA1", "TYMP")

#Create Matrices with just TMA Genes and Subject ID, Disease Category, and
Timepoint. (It's not necessary to keep the disease category and time point
but I did anyways)
NPX_Orig.TMA.healthy =
olink_data[which(olink_data$DiseaseCat==4),c(3,4,5,which(colnames(olink_data
) %in% TMA_Comprehensive_Panel))]
MISC_TPS.TMA = MISC_TPS[,c(1,2,3,which(colnames(MISC_TPS) %in%
TMA_Comprehensive_Panel))]


#Use Melt function in Reshape function to transform matrices into GGPLOT
compatible matrices
MISC_TPS.TMA.melted = melt(MISC_TPS.TMA, id =
c("SubjectID","Timepoint","DiseaseCat") )
NPX_Orig.TMA.healthy.melted = melt(NPX_Orig.TMA.healthy, id =
c("SubjectID","Timepoint","DiseaseCat") )

colnames(MISC_TPS.TMA.melted) =
c("Patient","Timepoint","diseaseCat","Gene","NPX")
colnames(NPX_Orig.TMA.healthy.melted) =
c("Patient","Timepoint","diseaseCat","Gene","NPX")



#Create custom X axis points for genes (will be needed for the individual
line plots per patient per gene)... This puts the X coordinates in the
right order of how you would want the data show according to the order of
the genes in the initial 'TMA_Comprehensive_Panel' vector declared in the
beginning of the script
X.1 = as.numeric(sapply(unique(MISC_TPS.TMA.melted$Gene),function(X) rep(c(
which(TMA_Comprehensive_Panel== X) -0.2, which(TMA_Comprehensive_Panel ==
X) +0.2),length(unique(MISC_TPS.TMA.melted$Patient)))))
X.1.boxplot =
as.numeric(sapply(unique(NPX_Orig.TMA.healthy.melted$Gene),function(X) rep(
which(TMA_Comprehensive_Panel == X)
,length(unique(NPX_Orig.TMA.healthy.melted$Patient)))))




#Attach X asis points to ggplot matrices
MISC_TPS.TMA.melted.X.1 = cbind(MISC_TPS.TMA.melted,X.1)
NPX_Orig.TMA.healthy.melted.X.1 =
cbind(NPX_Orig.TMA.healthy.melted,X.1.boxplot)
MISC_TPS.TMA.melted.X.1$Timepoint[which(MISC_TPS.TMA.melted.X.1$Timepoint=="1")]="Acute
"
MISC_TPS.TMA.melted.X.1$Timepoint[which(MISC_TPS.TMA.melted.X.1$Timepoint=="2")]="Covalescent
"

#Change Colnames ... just because I wanted to
colnames(MISC_TPS.TMA.melted.X.1) =
c("Patient","Timepoint","diseaseCat","Gene","NPX","Xcoord")
colnames(NPX_Orig.TMA.healthy.melted.X.1) =
```

```
c("Patient","Timepoint","diseaseCat","Gene","NPX","Xcoord")

# Make sure the factor levels of the the genes are in desired ordered
MISC_TPS.TMA.melted.X.1$Gene <- factor(MISC_TPS.TMA.melted.X.1$Gene,levels
= TMA_Comprehensive_Panel)
NPX_Orig.TMA.healthy.melted.X.1$Gene <-
factor(NPX_Orig.TMA.healthy.melted.X.1$Gene,levels =
TMA_Comprehensive_Panel)

#Create blank ggplot for data
PLOT.PLINE = ggplot(MISC_TPS.TMA.melted.X.1,aes(y = NPX, x=Xcoord))

#Add boxplot first so it aears behind the line plots
PLOT.PLINE = PLOT.PLINE + geom_boxplot(data =
NPX_Orig.TMA.healthy.melted.X.1,aes(y = NPX, x=Xcoord, fill=Gene),
outlier.shape=NA) + scale_fill_manual(values=rep(c("#BEBEBE80"),7)) +
theme(legend.position = "none")

##generate stats for every group
library(ggpubr)
anno_df = compare_means(NPX ~ Timepoint, group.by = "Gene", data =
MISC_TPS.TMA.melted.X.1, method="wilcox.test",paired=TRUE)

#Loop through each gene to add patient data per gene (this will prevent the
formation of a continuous line across the entire plot for each patient)

for(GENE in TMA_Comprehensive_Panel)
{
  PLOT.PLINE = PLOT.PLINE + geom_line(data =
MISC_TPS.TMA.melted.X.1[which(MISC_TPS.TMA.melted.X.1$Gene==GENE),],
mapping = aes(y = NPX, x=Xcoord, color=Patient))

  PLOT.PLINE = PLOT.PLINE +
scale_color_manual(values=rep(c("#000000"),length(X.1)))


  PLOT.PLINE = PLOT.PLINE + geom_point(data =
MISC_TPS.TMA.melted.X.1[which(MISC_TPS.TMA.melted.X.1$Gene==GENE),],
mapping = aes(y = NPX, x=Xcoord, color=Patient, shape=Timepoint))

  pval=anno_df$p.format[which(anno_df$Gene==GENE)]
  xval=MISC_TPS.TMA.melted.X.1[which(MISC_TPS.TMA.melted.X.1$Gene==GENE &
                                     MISC_TPS.TMA.melted.X.1$Timepoint ==
                                     "Acute"),"Xcoord"]

  PLOT.PLINE = PLOT.PLINE + geom_text(x=xval[1], y=12, label=pval, size=3)

}



PLOT.PLINE
#Change other features of plot
PLOT.PLINE = PLOT.PLINE + guides(color=FALSE, fill=FALSE )
PLOT.PLINE = PLOT.PLINE + theme_classic()
PLOT.PLINE = PLOT.PLINE + ylim(-5,13)
#Conveert X-axis to Gene Names: Be sure the factor order for the ggplot
matches the the order of genes in 'labels' vecotr
PLOT.PLINE = PLOT.PLINE + scale_x_continuous(breaks = 1:7, labels =
TMA_Comprehensive_Panel)

PLOT.PLINE = PLOT.PLINE + theme(axis.text.x = element_text(angle = 90,
vjust = 0.5, hjust = 1))

PLOT.PLINE = PLOT.PLINE + theme(axis.title.x = element_blank())
plot(PLOT.PLINE)
```


```{r}
##NPX expression of MAS associated genes

Panel2=c("IFNG", "CXCL9", "CD163", "IL2RA", "VSIG4", "HMOX1")

#Create Matrices with just TMA Genes and Subject ID, Disease Category, and
Timepoint. (It's not necessary to keep the disease category and timepoint
but I did anyways)
NPX_Orig.TMA.healthy =
olink_data[which(olink_data$DiseaseCat==4),c(3,4,5,which(colnames(olink_data
) %in% Panel2))]
MISC_TPS.TMA = MISC_TPS[,c(1,2,3,which(colnames(MISC_TPS) %in% Panel2))]


#Use Melt function in Reshape function to transform matrices into GGPLOT
compatible matrices
MISC_TPS.TMA.melted = melt(MISC_TPS.TMA, id =
c("SubjectID","Timepoint","DiseaseCat") )
NPX_Orig.TMA.healthy.melted = melt(NPX_Orig.TMA.healthy, id =
c("SubjectID","Timepoint","DiseaseCat") )

colnames(MISC_TPS.TMA.melted) =
```

```
c("Patient","Timepoint","diseaseCat","Gene","NPX")
colnames(NPX_Orig.TMA.healthy.melted) =
c("Patient","Timepoint","diseaseCat","Gene","NPX")



#Create custom X axis points for genes (will be needed for the individual
line plots per patient per gene)... This puts the X coordinates in the
right order of how you would want the data show according to the order of
the genes in the initial 'TMA_Comprehensive_Panel' vector declared in the
beginning of the script
X.1 = as.numeric(sapply(unique(MISC_TPS.TMA.melted$Gene),function(X) rep(c(
which(Panel2== X) -0.2, which(Panel2 == X)
+0.2),length(unique(MISC_TPS.TMA.melted$Patient)))))
X.1.boxplot =
as.numeric(sapply(unique(NPX_Orig.TMA.healthy.melted$Gene),function(X) rep(
which(Panel2 == X) ,length(unique(NPX_Orig.TMA.healthy.melted$Patient)))))




#Attach X asis points to ggplot matrices
MISC_TPS.TMA.melted.X.1 = cbind(MISC_TPS.TMA.melted,X.1)
NPX_Orig.TMA.healthy.melted.X.1 =
cbind(NPX_Orig.TMA.healthy.melted,X.1.boxplot)
MISC_TPS.TMA.melted.X.1$Timepoint[which(MISC_TPS.TMA.melted.X.1$Timepoint=="1")]="Acute
"
MISC_TPS.TMA.melted.X.1$Timepoint[which(MISC_TPS.TMA.melted.X.1$Timepoint=="2")]="Covalescent
"

#Change Colnames ... just because I wanted to
colnames(MISC_TPS.TMA.melted.X.1) =
c("Patient","Timepoint","diseaseCat","Gene","NPX","Xcoord")
colnames(NPX_Orig.TMA.healthy.melted.X.1) =
c("Patient","Timepoint","diseaseCat","Gene","NPX","Xcoord")

# Make sure the factor levels of the the genes are in desired ordered
MISC_TPS.TMA.melted.X.1$Gene <- factor(MISC_TPS.TMA.melted.X.1$Gene,levels
= Panel2)
NPX_Orig.TMA.healthy.melted.X.1$Gene <-
factor(NPX_Orig.TMA.healthy.melted.X.1$Gene,levels = Panel2)

#Create blank ggplot for data
PLOT.PLINE = ggplot(MISC_TPS.TMA.melted.X.1,aes(y = NPX, x=Xcoord))

#Add boxplot first so it aears behind the line plots

PLOT.PLINE = PLOT.PLINE + geom_boxplot(data =
NPX_Orig.TMA.healthy.melted.X.1,aes(y = NPX, x=Xcoord, fill=Gene),
outlier.shape=NA) +
  scale_fill_manual(values=rep(c("#BEBEBE80"),7)) + theme(legend.position =
"none")


##generate stats for every group
library(ggpubr)
anno_df = compare_means(NPX ~ Timepoint, group.by = "Gene", data =
MISC_TPS.TMA.melted.X.1, method="wilcox.test",paired=TRUE)

#Loop through each gene to add patient data per gene (this will prevent the
formation of a continuous line across the entire plot for each patient)
for(GENE in Panel2)
{
  PLOT.PLINE = PLOT.PLINE + geom_line(data =
MISC_TPS.TMA.melted.X.1[which(MISC_TPS.TMA.melted.X.1$Gene==GENE),],
mapping = aes(y = NPX, x=Xcoord, color=factor(Patient)))

  PLOT.PLINE = PLOT.PLINE +
scale_color_manual(values=rep(c("#000000"),length(X.1)))

  PLOT.PLINE = PLOT.PLINE + geom_point(data =
MISC_TPS.TMA.melted.X.1[which(MISC_TPS.TMA.melted.X.1$Gene==GENE),],
mapping = aes(y =

NPX, x=Xcoord, color=factor(Patient),

shape=Timepoint))

  pval=anno_df$p.format[which(anno_df$Gene==GENE)]
  xval=MISC_TPS.TMA.melted.X.1[which(MISC_TPS.TMA.melted.X.1$Gene==GENE &
                                      MISC_TPS.TMA.melted.X.1$Timepoint ==
                                      "Acute"),"Xcoord"]

  PLOT.PLINE = PLOT.PLINE + geom_text(x=xval[1], y=12, label=pval, size=3)

}

PLOT.PLINE
#Change other features of plot
PLOT.PLINE = PLOT.PLINE + guides(color=FALSE, fill=FALSE )
PLOT.PLINE = PLOT.PLINE + theme_classic()
```

````
PLOT.PLINE = PLOT.PLINE + ylim(-5,13)
#Conveert X-axis to Gene Names: Be sure the factor order for the ggplot
matches the the order of genes in 'labels' vecotr
PLOT.PLINE = PLOT.PLINE + scale_x_continuous(breaks = 1:6, labels = Panel2)

PLOT.PLINE = PLOT.PLINE + theme(axis.text.x = element_text(angle = 90,
vjust = 0.5, hjust = 1))

PLOT.PLINE = PLOT.PLINE + theme(axis.title.x = element_blank())


plot(PLOT.PLINE)
```


```{r}
##Heatmaps for MAS associated genes

#setup subset dataframe
heatmap.proteins = c("IFNG", "CXCL9", "CD163", "IL2RA", "VSIG4", "HMOX1")

#setup MYDATA data frame with categorical variables

HEATMAP <- olink_clinical
HEATMAP <- within(HEATMAP, {
  DiseaseCat <- factor(DiseaseCat, levels = 1:4, labels = c("MISC",
"Minimal", "Severe", "Healthy"))
})
HEATMAP[1,5:1,5]

prehm <- HEATMAP[,c("SubjectID", "DiseaseCat", heatmap.proteins)]
rownames(prehm) = with(prehm, paste(SubjectID, DiseaseCat, sep =":"))
hm <- prehm [, heatmap.proteins]

#set up disease cat dataframe
discat.df = data.frame ("Disease Category" = prehm$DiseaseCat)

rownames(discat.df) = rownames(prehm)

#Set up the colours. You need to call this the name of the clustering
column listed in the discat.df. Note that Ed named it Disease.Category
because he named it with a space.
ann_colors = list(
  Disease.Category = c(MISC = "#440154FF", Severe = "#31688EFF", Minimal =
"#35B779FF", Healthy = "#FDE725FF" )
)

#Draw Heat map
pheatmap(hm, fontsize = 10, color = inferno(30), annotation_row =
discat.df, annotation_colors = ann_colors, show_rownames = TRUE,
cluster_cols = FALSE, cellwidth = 12, annotation_names_row = FALSE)

olink_clinical$DiseaseCat=as.factor(olink_clinical$DiseaseCat)

ggscatter(olink_clinical, x = "IL10", y = "IL12B",
          add = "reg.line",                         # Add regression line
          conf.int = TRUE, # Add confidence interval
          color = "DiseaseCat",
          legend.title = "Disease Category",
          palette="jco") + stat_cor(aes(color = DiseaseCat),
label.y=c(10,9,8,7)) + xlab(NULL)
```


```{r}
##Identifying correlation between genes associated with vascular biology in
olink data

MY_COR = olink_clinical
cor_proteins = c("SC5B9", "PLA2G2A", "VWA1", "TYMP", "CALCA", "VEGFA",
"VEGFC",
                 "VEGFD", "FLT1", "FLT4", "EGFL7", "KDR", "PEAR1", "SELE",
"SERPINE1",
                 "SELP", "ESM1", "NOS3", "PECAM1", "TEK", "PDGFRA",
"PDGFRB", "PDGFA",
                 "GP1BA", "PDGFB", "GP6", "PDGFC", "LPCAT2", "VWF")
Cor_mat = cor(MY_COR[,cor_proteins], use="complete.obs")

#Function for p values in making a correlation matrix
# mat : is a matrix of data
# ... : further arguments to pass to the native R cor.test function
cor.mtest <- function(mat, ...) {
  mat <- as.matrix(mat)
  n <- ncol(mat)
  p.mat<- matrix(NA, n, n)
  diag(p.mat) <- 0
  for (i in 1:(n - 1)) {
    for (j in (i + 1):n) {
      tmp <- cor.test(mat[, i], mat[, j], ...)
      p.mat[i, j] <- p.mat[j, i] <- tmp$p.value
    }
````

```
  }
  colnames(p.mat) <- rownames(p.mat) <- colnames(mat)
  p.mat
}
p_mat = cor.mtest(MY_COR[,cor_proteins], use="complete.obs")

#Plot the correlation matrix; note that element addrect is telling it how
many squares to draw around everybody. To change the colour you need ot use
colorRampPalette to make an array of colours form viridis.
#I used inferno here because viridis and magma didn't look that nice

corrplot(Cor_mat, col = col5(50), order="hclust", tl.col = "black", addrect
= 7, p.mat = p_mat, sig.level = 0.05, insig = "blank", tl.cex = 0.7)
col5 <- colorRampPalette(inferno(5))
```

```{r}
##Pathway Analyses

library(pathfindR)

tp1=olink_data[which(olink_data$Timepoint==1),]
head(tp1)


## getting only the NPX columns
library(dplyr)
tp1_pca=select(tp1, -(1:4))
head(tp1_pca)

##changing names of disease categories

tp1$Diseasecat2=tp1$DiseaseCat
tp1$Diseasecat2[which(tp1$Diseasecat2 == "1")] = "misc"
tp1$Diseasecat2[which(tp1$Diseasecat2 == "2")] = "min_cov"
tp1$Diseasecat2[which(tp1$Diseasecat2 == "3")] = "sev_cov"
tp1$Diseasecat2[which(tp1$Diseasecat2 == "4")] = "healthy"
head(tp1$Diseasecat2)



##creating the thershold and identifying deferentially expressed genes
Pval_Threshold = 0.01
FC_Threshold = 2

##creating table for pathway analysis input

pathway_table_all = function(group1,group2){
  diff_exp_matrix_fc=apply(tp1_pca,MARGIN=2,function(X)
    mean(X[which(tp1$DiseaseCat==group1)])-
mean(X[which(tp1$DiseaseCat==group2)]))
  diff_exp_matrix_pval=apply(tp1_pca,MARGIN=2,function(X)

p.adjust(t.test(X[which(tp1$DiseaseCat==group1)],X[which(tp1$DiseaseCat==group2)])$p.value,method="BH",n=length(5:(ncol(tp1)
-1))))
  #creating table with only pval, logfc, and gene name
  DE_table=data.frame("pvalue"=diff_exp_matrix_pval,
"logFC"=diff_exp_matrix_fc, "gene"=names(diff_exp_matrix_fc))
  rownames(DE_table)=NULL
  #order table by pvalue
  order_table=DE_table[with(DE_table,order(pvalue)),]
  return(order_table)

}

##Create pathway analysis tables for all group comparisons
table.1.4=pathway_table_all("1","4")
table.2.4=pathway_table_all("2","4")
table.3.4=pathway_table_all("3","4")


#Running pathr function for group comparisons
pathr_output=function(gene_table, group1, group2){
  #Changing the table format to match what is needed in pathfindr
  colnames(gene_table)=c("adj.P.Val","logFC","Gene.symbol")
  pf_table=data.frame("Gene.symbol"=gene_table$Gene.symbol,
"logFC"=gene_table$logFC, "adj.P.val"=gene_table$adj.P.Val)
  #Creating a folder name to store all outputs of pathfindR
  dir_name2= paste("enrchiment",group1,".",group2,"kegg", sep="")
  output_df_kegg <- run_pathfindR(pf_table, p_val_threshold = 0.01,
output_dir = dir_name2)
  #looking at the top 20 enrichment terms
  enrichment_chart(result_df = output_df_kegg,
               top_terms = 20)

  return(output_df_kegg)
}

#running path function and storing tables in variables
```

```
pathr1.4=pathr_output(table.1.4, "1","4")
pathr2.4=pathr_output(table.2.4, "2","4")
pathr3.4=pathr_output(table.3.4,"3","4")
```

```{r}
term_gene_graph=function (result_df, num_terms = 10, layout = "stress",
use_description = FALSE,
    node_size = "num_genes")
{
    if (!is.numeric(num_terms) & !is.null(num_terms)) {
        stop("`num_terms` must either be numeric or NULL!")
    }
    if (!is.logical(use_description)) {
        stop("`use_description` must either be TRUE or FALSE!")
    }
    ID_column <- ifelse(use_description, "Term_Description",
        "ID")
    val_node_size <- c("num_genes", "p_val")
    if (!node_size %in% val_node_size) {
        stop("`node_size` should be one of ", paste(dQuote(val_node_size),
            collapse = ", "))
    }
    if (!is.data.frame(result_df))
        stop("`result_df` should be a data frame")
    necessary_cols <- c(ID_column, "lowest_p", "Up_regulated",
        "Down_regulated")
    if (!all(necessary_cols %in% colnames(result_df))) {
        stop(paste(c("All of", paste(necessary_cols, collapse = ", "),
            "must be present in `results_df`!"), collapse = " "))
    }
    if (!is.null(num_terms)) {
        if (nrow(result_df) < num_terms) {
            num_terms <- NULL
        }
    }
    result_df <- result_df[order(result_df$lowest_p, decreasing = FALSE),
        ]
    if (!is.null(num_terms)) {
        result_df <- result_df[1:num_terms, ]
    }
    graph_df <- data.frame()
    for (i in base::seq_len(nrow(result_df))) {
        up_genes <- unlist(strsplit(result_df$Up_regulated[i],
            ", "))
        down_genes <- unlist(strsplit(result_df$Down_regulated[i],
            ", "))
        genes <- c(up_genes, down_genes)
        for (gene in genes) {
            graph_df <- rbind(graph_df, data.frame(Term = result_df[i,
                ID_column], Gene = gene))
        }
    }
    up_genes <- lapply(result_df$Up_regulated, function(x)
unlist(strsplit(x,
        ", ")))
    up_genes <- unlist(up_genes)
    g <- igraph::graph_from_data_frame(graph_df, directed = FALSE)
    cond_term <- names(igraph::V(g)) %in% result_df[, ID_column]
    cond_up_gene <- names(igraph::V(g)) %in% up_genes
    igraph::V(g)$type <- ifelse(cond_term, "term", ifelse(cond_up_gene,
        "up", "down"))
    if (node_size == "num_genes") {
        sizes <- igraph::degree(g)
        sizes <- ifelse(igraph::V(g)$type == "term", sizes, 2)
        size_label <- "# proteins"
    }
    else {
        idx <- match(names(igraph::V(g)), result_df[, ID_column])
        sizes <- -log10(result_df$lowest_p[idx])
        sizes[is.na(sizes)] <- 2
        size_label <- "-log10(p)"
    }
    igraph::V(g)$size <- sizes
    igraph::V(g)$label.cex <- 0.4
    igraph::V(g)$frame.color <- "gray"
    igraph::V(g)$color <- ifelse(igraph::V(g)$type == "term",
        "#E5D7BF", ifelse(igraph::V(g)$type == "up", "hotpink1",
            "orange"))

    p <- ggraph::ggraph(g, layout = layout)
    p <- p + ggraph::geom_edge_link(alpha = 0.6, colour = "darkgrey")
    p <- p + ggraph::geom_node_point(ggplot2::aes_(color = ~I(color),
        size = ~size))
    p <- p + ggplot2::scale_size(range = c(5, 10), breaks =
round(seq(round(min(igraph::V(g)$size)),
        round(max(igraph::V(g)$size)), length.out = 4)), name = size_label)
    p <- p + ggplot2::theme_void()
    p <- p + ggraph::geom_node_text(ggplot2::aes_(label = ~name),
        nudge_y = 0.2, repel = TRUE, size=2)
```

````
    p <- p + ggplot2::scale_colour_manual(values =
unique(igraph::V(g)$color),
        name = NULL, labels = c("enriched term", "up-regulated protein",
            "down-regulated protein"))

    if (is.null(num_terms)) {
        p <- p + ggplot2::ggtitle("Term-Protein Graph")
    }
    else {
        p <- p + ggplot2::ggtitle("Term-Protein Graph", subtitle =
paste(c("Top",
            num_terms, "terms"), collapse = " "))
    }
    p <- p + ggplot2::theme(plot.title = ggplot2::element_text(hjust =
0.2),
        plot.subtitle = ggplot2::element_text(hjust = 0.2))

    return(p)
}
```


```{r}
#Creating graphs with specific terms included

terms_1.4=c("Cytokine-cytokine receptor interaction", "Apoptosis", "TNF
signaling pathway", "Chemokine signaling pathway")
terms_1.4v2=c("Apoptosis", "TNF signaling pathway", "Chemokine signaling
pathway")
ind_1.4=which(pathr1.4$Term_Description %in% terms_1.4v2)
gene_graph.1.4=pathr1.4[ind_1.4[1:3],]
term_gene_graph(result_df = gene_graph.1.4,use_description = TRUE, layout =
"fr")

#graph for 2 vs 4
terms_2.4=c("IL-17 signaling pathway", "Cytokine-cytokine receptor
interaction", "Chemokine signaling pathway", "TNF signaling pathway" )
ind_2.4=which(pathr2.4$Term_Description %in% terms_2.4)
gene_graph.2.4=pathr2.4[ind_2.4,]

#saving image of plot
term_gene_graph(result_df = gene_graph.2.4,use_description = TRUE, layout =
"fr")

#graph 3 vs 4
##cytokine-cytokine, chemokine signaling, bacterial
terms_3.4=c( "Cytokine-cytokine receptor interaction", "Estrogen signaling
pathway", "Arginine and proline metabolism" )
ind_3.4=which(pathr3.4$Term_Description %in% terms_3.4)
gene_graph.3.4=pathr3.4[ind_3.4,]
term_gene_graph(result_df = gene_graph.3.4, use_description = TRUE, layout=
"fr")



```






## Including Plots

You can also embed plots, for example:

```{r pressure, echo=FALSE}
plot(pressure)
```

Note that the `echo = FALSE` parameter was added to the code chunk to
prevent printing of the R code that generated the plot.
````
````
