# Supplementary material for: Proteomic profiling of MIS-C patients indicates heterogeneity relating to interferon gamma dysregulation and vascular endothelial dysfunction
Source: Nat Commun. 2021 Dec 10;12:7222. doi: 10.1038/s41467-021-27544-6 (PMC8664884; doi:10.1038/s41467-021-27544-6)
Supplement: Supplementary file 3 — Description of Additional Supplementary Files [file 41467_2021_27544_MOESM3_ESM.pdf]

Description of Additional Supplementary Files

File Name: Supplementary Data 1
Description: Lists of differentially expressed proteins (DEP) between patients with MIS-C, Severe COVID-19, Minimal disease and healthy controls.

File Name: Supplementary Data 2
Description: Lists of ranked pathways corresponding to pathway analysis presented in Figure 2.
