# Supplementary material for: Proteomic profiling of MIS-C patients indicates heterogeneity relating to interferon gamma dysregulation and vascular endothelial dysfunction
Source: Nat Commun. 2021 Dec 10;12:7222. doi: 10.1038/s41467-021-27544-6 (PMC8664884; doi:10.1038/s41467-021-27544-6)
Supplement: Supplementary file 6 — Reporting Summary [file 41467_2021_27544_MOESM6_ESM.pdf]

nature research

Corresponding author(s): David T. Teachey

Last updated by author(s): Oct 27, 2021

Reporting Summary

Nature Research wishes to improve the reproducibility of the work that we publish. This form provides structure for consistency and transparency in reporting. For further information on Nature Research policies, see Authors & Referees and the Editorial Policy Checklist.

Statistics

For all statistical analyses, confirm that the following items are present in the figure legend, table legend, main text, or Methods section.

n/a	Confirmed	
☐	☒	The exact sample size (*n*) for each experimental group/condition, given as a discrete number and unit of measurement
☒	☐	A statement on whether measurements were taken from distinct samples or whether the same sample was measured repeatedly
☐	☒	The statistical test(s) used AND whether they are one- or two-sided<br>*Only common tests should be described solely by name; describe more complex techniques in the Methods section.*
☐	☒	A description of all covariates tested
☐	☒	A description of any assumptions or corrections, such as tests of normality and adjustment for multiple comparisons
☐	☒	A full description of the statistical parameters including central tendency (e.g. means) or other basic estimates (e.g. regression coefficient) AND variation (e.g. standard deviation) or associated estimates of uncertainty (e.g. confidence intervals)
☐	☒	For null hypothesis testing, the test statistic (e.g. *F*, *t*, *r*) with confidence intervals, effect sizes, degrees of freedom and *P* value noted<br>*Give P values as exact values whenever suitable.*
☒	☐	For Bayesian analysis, information on the choice of priors and Markov chain Monte Carlo settings
☐	☒	For hierarchical and complex designs, identification of the appropriate level for tests and full reporting of outcomes
☐	☒	Estimates of effect sizes (e.g. Cohen's *d*, Pearson's *r*), indicating how they were calculated

Our web collection on statistics for biologists contains articles on many of the points above.

Software and code

Policy information about availability of computer code

Data collection	RedCAP software was used for data collection of clinical data (version 11.2.2)
Data analysis	All statistical analyses were performed in R (version 4.0.4) using RStudio (RStudio, PBC, Boston, MA). Flow cytometry analysis was performed in FlowJo (Treestar, version 10.6.2).

For manuscripts utilizing custom algorithms or software that are central to the research but not yet described in published literature, software must be made available to editors/reviewers. We strongly encourage code deposition in a community repository (e.g. GitHub). See the Nature Research guidelines for submitting code & software for further information.

Data

Policy information about availability of data

All manuscripts must include a data availability statement. This statement should provide the following information, where applicable:

- Accession codes, unique identifiers, or web links for publicly available datasets
- A list of figures that have associated raw data
- A description of any restrictions on data availability

The NPX data generated in this study have been deposited in the PRIDE database under accession code PXD029375 [http://www.ebi.ac.uk/pride]. The NPX data generated in this study are provided in the Supplementary Information/Source Data file. The Source Code is available in the Supplementary Information/Source Code file. We also used the KEGG pathway database available here (https://www.genome.jp/kegg/pathway.html).

Field-specific reporting

Please select the one below that is the best fit for your research. If you are not sure, read the appropriate sections before making your selection.

☒ Life sciences ☐ Behavioural & social sciences ☐ Ecological, evolutionary & environmental sciences

For a reference copy of the document with all sections, see nature.com/documents/nr-reporting-summary-flat.pdf

Life sciences study design

All studies must disclose on these points even when the disclosure is negative.

Sample size	Samples size was chosen based on the availability of patient samples for a rare pediatric condition.
Data exclusions	No data were excluded from analyses.
Replication	We replicated data from O-link analyses by cytokine analysis in our clinical and research laboratory. These data are presented in supplemental figure 1. Data from our clinical and research labs was available on 76/88 of the patients studied. All replication attempts were successful.
Randomization	This was a cohort study comparing patients with different categories of disease so randomization was not possible.
Blinding	The O-link analytic process was blinded to the disease category of the patients. Analysis was completed based on disease categories and thus blinding was not relevant.

Reporting for specific materials, systems and methods

We require information from authors about some types of materials, experimental systems and methods used in many studies. Here, indicate whether each material, system or method listed is relevant to your study. If you are not sure if a list item applies to your research, read the appropriate section before selecting a response.

Materials & experimental systems

n/a	Involved in the study	
☒	☐	Antibodies
☒	☐	Eukaryotic cell lines
☒	☐	Palaeontology
☒	☐	Animals and other organisms
☐	☒	Human research participants
☒	☐	Clinical data

Methods

n/a	Involved in the study	
☒	☐	ChIP-seq
☐	☒	Flow cytometry
☒	☐	MRI-based neuroimaging

Human research participants

Policy information about studies involving human research participants

Population characteristics	Patients admitted to the Children's Hospital of Philadelphia with a SARS-CoV-2 infection or with MIS-C were included in this study. Age, gender and disease category are presented in Supplemental Table S1.
Recruitment	All patients with SARS-CoV-2 infection who were admitted during the study period were eligible for enrollment. As pediatric patients were the focus of this study blood draws performed exclusively for study purposes were not allowed. Every effort was made to offer enrollment in the biobanking protocol to all eligible patients. Patients that had very brief admissions or who did not have labs drawn during their admission for clinical reasons were not included as no blood sample could be obtained from those patients. Thus, patients with minimal SARS-CoV-2 infection symptoms were also likely to have other conditions.
Ethics oversight	This study was conducted in accordance with the Declaration of Helsinki and received approval from the Institutional Review Board (IRB) at CHOP.

Note that full information on the approval of the study protocol must also be provided in the manuscript.

Flow Cytometry

Plots

Confirm that:

- [ ] The axis labels state the marker and fluorochrome used (e.g. CD4-FITC).
- [ ] The axis scales are clearly visible. Include numbers along axes only for bottom left plot of group (a 'group' is an analysis of identical markers).
- [ ] All plots are contour plots with outliers or pseudocolor plots.
- [ ] A numerical value for number of cells or percentage (with statistics) is provided.

Methodology

Sample preparation	Primary analysis of flow cytometry data is reported in Vella et al. Sci Immunol 2021. We provided a comparison between our proteomic data and these previously reported data. Thus, flow cytometry data is reported in this report as correlative measures with proteomic data as the main focus of the paper is on the proteomic data.
Instrument	*Identify the instrument used for data collection, specifying make and model number.*
Software	*Describe the software used to collect and analyze the flow cytometry data. For custom code that has been deposited into a community repository, provide accession details.*
Cell population abundance	*Describe the abundance of the relevant cell populations within post-sort fractions, providing details on the purity of the samples and how it was determined.*
Gating strategy	*Describe the gating strategy used for all relevant experiments, specifying the preliminary FSC/SSC gates of the starting cell population, indicating where boundaries between "positive" and "negative" staining cell populations are defined.*

- [ ] Tick this box to confirm that a figure exemplifying the gating strategy is provided in the Supplementary Information.
